# Enhancing grapefruit resistance to green mold through synergistic effects of bamboo vinegar and *Cryptococcus laurentii*

**DOI:** 10.1016/j.fochx.2026.104540

**Published:** 2026-09-29

**Authors:** Ruiqi Peng, Han Ye, Chunhua Wu, Xiahong He, Fang Wang, Jia Deng

**Affiliations:** a Forestry College, Southwest Forestry University, Kunming 650224, PR China; b Key Laboratory of State Forestry and Grassland Administration on Highly-Efficient Utilization of Forestry Bioass Resources in Southwest China, College of Materials and Chemical Engineering, Southwest Forestry University, Kunming 650224, PR China; c Yunnan Provincial Key Laboratory for Conservation and Utilization of In-Forest Resource, Southwest Forestry University, Kunming 650224, PR China; d Key Laboratory of State Forestry Administration on Biodiversity Conservation in Southwest China, Southwest Forestry University, Kunming 650224, PR China; e Key Laboratory for Forest Resources Conservation and Use in the Southwest Mountains of China, Ministry of Education, Southwest Forestry University, Kunming 650224, PR China

**Keywords:** Grapefruit, Bamboo vinegar, *Cryptococcus laurentii*, Defensive response, Fruit quality

## Abstract

Green mold (*Penicillium* spp.) is a major postharvest disease of grapefruit. Optimal concentrations of bamboo vinegar (BV) and *Cryptococcus laurentii* were screened in vitro, and their individual and combined efficacy evaluated. BV (1%, 121 °C-sterilized) plus 1 × 10^7^ CFU mL^−1^*C. laurentii* (BV–*C. laurentii*) significantly inhibited spore germination and germ tube elongation in *Penicillium* spp., disrupted *P. digitatum* hyphal cell wall and membrane integrity, and increased extracellular chitinase and β-1,3-glucanase activities by 1.05- and 1.22-fold over *C. laurentii* alone at 48 h while reducing malondialdehyde and reduced glutathione. BV–*C. laurentii* also activated fruit innate defense, with phenylalanine ammonia-lyase and polyphenol oxidase activities 1.03–1.44-fold higher than CK and total phenol and flavonoid contents 20.22% and 19.73% higher on day 28, without adversely affecting total soluble solids, titratable acidity, ascorbic acid or reducing sugar. Thus, BV–*C. laurentii* effectively controls green mold and delays quality deterioration in grapefruit.

## Highlights

• Bamboo vinegar and *Cryptococcus laurentii* inhibits *Penicillium* in grapefruit.
• 1% BV and 1 × 10^7^ CFU mL^−1^*C. laurentii* are the optimal antibacterial concentrations.
• The combined treatment up-regulate CHI, GLU and GR activities and lower MDA and GSH.
• The combined treatment enhanced defense-related enzyme activity in grapefruit.
• The combined treatment maintains grapefruit fruit storage quality.

## 1 Introduction

Grapefruit (*Citrus paradisi* Macf.), as a perennial evergreen fruit tree of the *Citrus* genus in the Rutaceae family, it is a natural hybrid of pomelo and sweet orange (Devi et al., 2024). Its pulp is rich in proteins, organic acids, and minerals such as iron and calcium. Renowned for its low sugar content and high vitamin C levels, grapefruit offers significant nutritional value and boasts benefits like beauty and skin-enhancing effects (Garcia-Lor et al., 2021). Moreover, the bioactive compounds present in grapefruit peel and seeds, such as flavonoids, exhibit antioxidant and antimicrobial properties, making them widely applicable in medical research (Ciriminna et al., 2025). However, fresh grapefruit is highly susceptible to pathogen infection during postharvest transportation, storage, and marketing. Among these, fruit rot caused by *Penicillium* is the most detrimental, leading to fruit deterioration and even decay, resulting in substantial postharvest losses and severe economic implications (Strano et al., 2022). Currently, physical and chemical methods are employed to suppress diseases and preserve fruit quality during postharvest storage and transportation of grapefruit, aiming to minimize losses. Nevertheless, physical approaches such as low-temperature storage, irradiation, and heat treatment entail high costs and complex operational procedures (Lisboa et al., 2024), while the use of chemical fungicides not only causes severe environmental pollution but also poses significant health risks to consumers due to their long-term persistence (Zhou et al., 2022). Therefore, it's of great significance to seek out safer and more eco-friendly methods for controlling postharvest diseases in grapefruits. Employing antagonistic yeasts as a biological control measure presents a promising, non-toxic substitute for chemical fungicides. Antagonistic yeasts are generally less effective in suppressing diseases compared to chemical fungicides. Consequently, there is a pressing need to develop strategies to enhance the biocontrol effectiveness of yeast-based approaches (Edo et al., 2025).

Yeast represents a significant group of antagonistic microorganisms, characterized by rapid proliferation and strong adaptability, serving as a primary microbial agent for postharvest fruit preservation (He et al., 2024). As a yeast with biocontrol potential, *Cryptococcus laurentii* has garnered significant attention from researchers in recent years. Li et al. (2019) discovered that *C. laurentii* was capable of disrupting the hyphal structure of *P. italicum*, thereby enhancing the control efficacy against citrus blue mold disease. Moreover, *C. laurentii* activated the activities of superoxide dismutase (SOD), phenylalanine ammonia-lyase (PAL), and catalase (CAT) in snap beans, while reducing malondialdehyde (MDA) content, these mechanisms collectively bolster plant disease resistance and delay plant senescence (Feng et al., 2021). Integrating protectants or inducers into the culture medium can substantially improve the biocontrol effectiveness of resistant yeasts. Xylitol has been demonstrated to decrease gray mold and blue mold occurrence in apples by elevating the expression levels of antioxidant genes (*TrxR*, *POD*) and ATP levels in *M. guilliermondii* (Zhang et al., 2025). Huang et al. (2022) observed that a 2% sodium glutamate solution enhanced the inhibitory effects of *R. paludigenum* against *P. expansum*, effectively reducing lesion size in pear fruit affected by green mold. Our research team has also conducted relevant studies in the earlier stages. Wu et al. (2024) found that carboxymethyl chitosan enhanced *C. laurentii* biofilm formation, increasing its biocontrol effectiveness against *P. expansum* in grapefruits. Furthermore, *C. laurentii* induced by melatonin was found to suppress grapefruit blue mold by regulating cell wall and membrane lipid metabolism, while also effectively preserving the nutritional quality of grapefruits and delaying their senescence and softening (Zhou et al., 2025). Unlike existing elicitors such as chitosan and melatonin, which function primarily by strengthening host physical barriers or regulating endogenous defense pathways in plants, bamboo vinegar-a byproduct derived from bamboo pyrolysis and rich in phenolic compounds and organic acids-possesses both direct antimicrobial activity and natural antioxidant properties. Distinct from chitosan, which mainly relies on its film-forming physical barrier effect, and melatonin, which predominantly modulates the endogenous antioxidant pathways in harvested fruits, bamboo vinegar-mediated yeast induction achieves a triple synergistic effect: direct pathogen inhibition, enhanced yeast vitality, and activated fruit defense responses. This multi-mode working mechanism fundamentally differs from the single-function induction systems reported in previous studies (Yu et al., 2018). For example, bamboo vinegar has been shown to alleviate oxidative damage in *Perilla frutescens* caused by antioxidant system disruption under cadmium (Cd) stress, thereby improving the plant's self-repair capacity (Li et al., 2024). Shi et al. (2019) found that the combined application of bamboo vinegar and peach gum enhanced the activity of defense enzymes in blueberries without compromising their quality, while also reducing decay caused by blue and gray mold diseases. These findings suggest that bamboo vinegar holds promise as a complementary agent in postharvest disease management.

Currently, research on the application of bamboo vinegar-induced *C. laurentii* in influencing the quality changes of grapefruits during storage remains relatively scarce, particularly concerning the underlying mechanisms at the physiological and biochemical mechanisms that drive the induction of plant defense mechanisms. Therefore, this study employed bamboo vinegar-induced *C. laurentii* to explore the following objectives: 1) To determine the optimal antibacterial concentrations of bamboo vinegar and *C. laurentii* against *P. italicum*, *P. digitatum*, and *P. expansum*; 2) To assess the inhibitory efficacy of individual or combined treatments with bamboo vinegar and *C. laurentii* against green mold disease in grapefruits; 3) To investigate alterations in the activities of defense-related enzymes and the accumulation of defense-related substances in grapefruits following individual or combined treatments with bamboo vinegar and *C. laurentii*; 4) To analyze changes in the storage quality of grapefruits after individual or combined treatments.

## 2 Material and methods

### 2.1 Fruits, pathogens, and antagonists

The grapefruit cultivar employed in this experiment was ‘Rio Red’ grapefruit, uniformly procured from a grapefruit orchard located in Jiasa Town, Xinping County, Yunnan Province, China. Grapefruit fruits were carefully selected based on uniform size, consistent maturity, similar coloration, and the absence of pest infestations or mechanical injuries. Prior to laboratory processing, the fruits were initially rinsed with sterile water, followed by wiping with a 75% alcohol solution, and subsequently allowed to air-dry naturally before being set aside for further use.

The fungal strains *P. digitatum*, *P. italicum* and *P. expansum* were isolated from infected grapefruits. These strains were cultured on Potato Dextrose Agar (PDA) medium, and single colonies were transferred to PDB medium for shaking cultivation at 150 rpm for 48 h to prepare a spore suspension with a concentration of 1 × 10^5^ CFU mL^−1^, which was then stored for subsequent use. All three strains are currently preserved in the Key Laboratory of Biodiversity Conservation in Southwest China, Southwest Forestry University.

*C. laurentii* (ATCC 18803) was purchased from the Guangdong Institute of Microbiology, and the strain is currently preserved and maintained for experimental research in the Key Laboratory of Southwest Biodiversity Conservation, Southwest Forestry University. Bamboo vinegar was procured from Suichang Wutailang Bamboo Charcoal Co., Ltd.

### 2.2 Preliminary screening of antifungal activity of different concentrations of *C. laurentii* against *Penicillium*

A single colony of *C. laurentii* was inoculated into Yeast Extract-Malt Extract (YM) medium and cultivated on a shaker at 28 °C with a rotation speed of 120 rpm for 72 h. Subsequently, three concentrations of yeast cell suspensions (10^7^, 10^6^, and 10^5^ CFU mL^−1^) were prepared using distilled water. Following the method described by Wang et al. (2019), the germination tube length and germination rate of conidia from *P. digitatum*, *P. expansum*, and *P. italicum* were evaluated. Given the significant differences in spore germination rates among the three *Penicillium* spp., differentiated dynamic observation time points were set for each strain. Through comparative analysis of the germination rate and germ tube length at each time point, the final observation time points were determined as 10, 12.5 and 19 h for *P. digitatum*, 6, 7 and 9 h for *P. expansum*, and 7, 8 and 9 h for *P. italicum*. For the experimental setup, 200 mL sterile conical flasks were used, each containing 48 mL of PDB medium. To each flask, 1 mL of the aforementioned *C. laurentii* cell suspensions at the three different concentrations was added. Sterile water served as the control. Additionally, 1 mL of a *P. digitatum* spore suspension was introduced into each flask. The cultures were then incubated on a shaker at 28 °C and 120 rpm. Samples were taken at 10, 12.5, and 19 h post-inoculation. For each sample, approximately 100 spores were randomly observed under an optical microscope. The spore germination rate and the length of the germ tubes were then documented. The same procedure was applied to evaluate the inhibitory effects of different concentrations of *C. laurentii* on *P. expansum* and *P. italicum*, with sampling times adjusted to 6, 7, and 9 h for *P. expansum*, and 7, 8, and 9 h for *P. italicum*, respectively. Each treatment was replicated three times, and the experiment was conducted independently on two separate occasions to ensure the results' reliability and reproducibility.

### 2.3 Preliminary screening of antifungal activity of different concentrations of bamboo vinegar against *Penicillium*

PDB media with bamboo vinegar volume fractions of 0%, 0.1%, 0.5%, and 1% were prepared and sterilized at 121 °C for 20 min before use. For each experimental setup, 49 mL of PDB medium with the specified bamboo vinegar concentrations was transferred into a 200 mL conical flask. Flasks received 1 mL *P. digitatum* spore suspension and were shaken at 28 °C, 120 rpm. Samples were collected at 10, 12.5, and 19 h post-inoculation. For each sample, around 100 spores were randomly examined using an optical microscope todocument the germination rate and germ tube length. The experimental procedure to assess the inhibitory effects of varying bamboo vinegar concentrations on *P. expansum* and *P. italicum* was conducted with sampling times set at 6, 7, and 9 h for *P. expansum*, and 7, 8, and 9 h for *P. italicum*. To ensure robustness and reproducibility of findings, each experimental treatment was performed in triplicate, and the entire study was independently replicated twice.

### 2.4 Effects of different concentrations of bamboo vinegar on the growth of *C. laurentii*

Bamboo vinegar with varying volume fractions (0%, 0.2%, 0.4%, 0.5%, 0.6%, 0.8%, and 1%) were prepared and incorporated into YM liquid medium. The medium containing bamboo vinegar was then sterilized at 121 °C for 20 min and set aside for subsequent use. A volume of 1 mL of the prepared yeast suspension with a concentration of 1 × 10^7^ CFU mL^−1^ was added to 49 mL of the YM liquid medium containing different concentrations of bamboo vinegar. The cultures were incubated in a shaking incubator at 28 °C with a shaking speed of 120 rpm. Samples were collected at 6, 12, 24, and 48 h of incubation. The viable count of *C. laurentii* cells was determined using the spread plate method with serial dilutions. This approach was employed to evaluate the impact of different concentrations of bamboo vinegar on the growth of *C. laurentii*.

### 2.5 Inhibitory effect of *C. laurentii* treated with bamboo vinegar on *Penicillium*

Based on the results from 2.2, 2.3, 2.4, the optimal concentrations for inhibiting *Penicillium* were determined to be 1 × 10^7^ CFU mL^−1^ of *C. laurentii* and 1% (*v*/v) bamboo vinegar. To prepare the experimental materials, YM liquid media containing 1% (*v*/v) bamboo vinegar and regular YM liquid media were formulated and sterilized for subsequent use. Single colonies of *C. laurentii* of uniform size were then introduced into the prepared media and cultured on an orbital shaker at 28 °C with 120 rpm agitation for 48 h. The resulting fermentation broth was adjusted to a concentration of 1 × 10^7^ CFU mL^−1^. For the inhibitory effect assessment against *P. digitatum*, 1 mL of the 1 × 10^5^ CFU mL^−1^ *P. digitatum* suspension and 1 mL of the 1 × 10^7^ CFU mL^−1^ fermentation broth were added to 48 mL of PDB, then the sample maintained at 28 °C with 120 rpm agitation. Samples were collected at 10, 12.5, and 19 h post-inoculation. For each sample, approximately 100 spores were randomly observed under an optical microscope, and the spore germination rate and germ tube length were recorded at different time points using a hemocytometer. The experimental procedures for evaluating the inhibitory effects of bamboo vinegar-induced *C. laurentii* on *P. expansum* and *P. italicum* were identical to those described above, with the exception of the sampling times. For *P. expansum*, samples were taken at 6, 7, and 9 h post-inoculation, while for *P. italicum*, samples were collected at 7, 8, and 9 h post-inoculation. Each treatment group consisted of three biological replicates, and the complete experimental protocol was conducted twice on separate occasions to validate result reproducibility.

### 2.6 Effects of BV-*C. laurentii* on the cell wall integrity and membrane permeability of *P. digitatum*

After being cultured for 5 days, *P. digitatum* was punched into 5 mm mycelial disks with a cork borer and placed into 25 mL of PDA liquid medium. The samples were then incubated on a shaker at 28 °C and 120 rpm for 48 h. Following incubation, the mycelial disks were rinsed with sterile water and then transferred into 4 mL of a BV-*C. laurentii* fermentation broth (1 × 10^7^ CFU mL^−1^) that had been cultured for 48 h. As a control, mycelial blocks were placed in sterile water. Subsequently, all samples underwent incubation on a shaker set at 120 rpm and 28 °C. Sampling was conducted at 12, 24, and 48 h post-incubation, with three replicates for each treatment. The integrity of *P. digitatum* mycelial cell walls was assessed using the Calcofluor White (CFW) staining method, as outlined by Reymick et al. (2022). Additionally, the membrane permeability of the cells was assessed using Propidium iodide (PI) staining.

### 2.7 Tolerance of bamboo vinegar-induced *C. laurentii* to different concentrations of H_2_O_2_

According to Watanabe et al. (2022), the fermentation broth co-cultured with 1% (*v*/v) bamboo vinegar and *C. laurentii* for 48 h was prepared as outlined in Section 2.5, the mixture was adjusted to a concentration of 1 × 10^7^ CFU mL^−1^. Subsequently, 100 mL of the fermentation broth was transferred into conical flasks. The bacterial suspension in each flask was then adjusted to contain H₂O₂ at final volume fractions of 0%, 0.5%, 2%, and 5%, respectively. These flasks were incubated on a shaker at 28 °C and 120 rpm for 30 min, after which the fermentation broth was collected. The collected fermentation broth was diluted 10^6^-fold and then spread evenly onto YM solid media. The plates were incubated at 28 °C for 48 h, after which colony counts were used to calculate the viable cell concentration per milliliter of the original bacterial suspension, accounting for the dilution factor.

### 2.8 Determination of oxidative stress-related indicators and defense enzyme activities in *C. laurentii* induced by bamboo vinegar

To prepare the fermentation broth for evaluation, a 1% bamboo vinegar solution was co-cultured with *C. laurentii* for 48 h under the conditions specified in Section 2.5. This fermentation broth was subsequently utilized to assess the oxidative stress-related indicators and defensive enzyme activities of *C. laurentii*. The activities of CAT and glutathione reductase (GR) in the fermentation broth were measured using CAT and GR assay kits (Suzhou Mengxi Biomedical Technology Co., Ltd.). A single unit of CAT activity was characterized as the quantity of CAT necessary to facilitate the breakdown of 1 nmol of H₂O₂ per milliliter of the fermentation broth each minute. Similarly, one unit of GR activity represented the amount of GR necessary to catalyze the reduction of 1 nmol of NADPH per milliliter of fermentation broth per minute. The activity of chitinase (CHI) was defined as one enzyme activity unit corresponding to the catalytic generation of 1 mg *N*-acetyl-D-glucosamine per milliliter of fermentation broth within 1 h. Meanwhile, the activity of glucanase (GLU) was quantified as one enzyme activity unit for every 1 mg of reducing sugar released per milliliter of fermentation broth (Wang et al., 2021). The determination of MDA content was carried out following the approach outlined by Ganhão et al. (2011). The MDA value represented the concentration of MDA in the fermentation broth, expressed in nmol per milliliter. The measurement of reduced glutathione (GSH) content was conducted in accordance with the method described by Nishimoto et al. (2017). The GSH value indicated the concentration of reduced glutathione in the fermentation broth, reported in micromoles per milliliter. Each treatment involved three replicates, and the whole experiment was carried out twice to guarantee the reliability and repeatability of the findings.

### 2.9 Effects of different treatments on the incidence and lesion diameter of green mold disease in grapefruits

A 48-h co-cultured fermentation broth was prepared by incubating 1% bamboo vinegar together with *C. laurentii*. The concentration of *C. laurentii* in the fermentation broth was standardized to 1 × 10^7^ CFU mL^−1^ using a hemocytometer and reserved for subsequent use. Sterilized and dried grapefruits were submerged for 3 min in one of the subsequent solutions: 1) distilled water (control group, CK); 2) a fermentation broth with *C. laurentii* at 1 × 10^7^ CFU mL^−1^ (*C. laurentii*); 3) 1% bamboo vinegar (1% BV); and 4) a 48-h co-cultured fermentationbroth of 1% BV and *C. laurentii* (BV-*C. laurentii*). After immersion, the fruits were air-dried naturally. Each grapefruit wound received 5 uL of a *P. digitatum* spore suspension at 1 × 10^7^ CFU mL^−1^. The dried grapefruits were stored in sterile plastic boxes at 22 °C and 85% relative humidity. Daily assessments were conducted to evaluate the disease incidence and lesion diameter of the grapefruits. The same experimental procedures were applied for *P. expansum* and *P. italicum*. Each treatment group consisted of 9 grapefruits, and the experiment was repeated twice.

### 2.10 Effects of bamboo vinegar, *C. laurentii*, and their combined treatment on disease-resistance enzymes and compounds in grapefruits

The grapefruit treatment method was consistent with that described in Section 2.4. Grapefruits were treated individually with *C. laurentii* fermentation broth, bamboo vinegar, or a combination of both. Samples were gathered on days 7, 14, 21, and 28 after treatment and promptly frozen in liquid nitrogen for storage. Subsequently, the activities of polyphenol oxidase (PPO), PAL, GLU, and CHI, as well as the contents of flavonoids and total phenols, were measured. The levels of PPO, PAL, GLU, and CHI activities were measured using relevant assay kits supplied by Suzhou Mengxi Biomedical Technology Co., Ltd. The activity of PPO was expressed as the amount of enzyme required per gram of fresh weight to produce an increase of 1 absorbance unit at 420 nm per minute. Similarly, the activity of PAL was defined as the enzyme quantity per gram of fresh weight needed to cause an increase of 0.01 absorbance unit at 290 nm per minute. GLU activity was indicated as the enzyme amount per gram of fresh weight that catalyzes the generation of 1 mol of glucose per hour. In contrast, CHI activity was measured as the enzyme quantity per gram of fresh weight required to yield 1 mol of *N*-acetyl-D-glucosamine per hour. All enzyme activities were reported in units per gram of fresh weight (U g^−1^). To determine the total phenolic and flavonoid contents, the procedure outlined by Huang et al. (2023) was employed. Specifically, 1 g of the sample was placed in a mortar, to which 2 mL of pre-cooled 1% (*v*/v) HCl-methanol solution was added. The sample was thoroughly ground to a homogenous state and then transferred to a 10 mL centrifuge tube. An additional 2 mL of 1% HCl-methanol solution was used to rinse the mortar, and the rinsate was also transferred to the centrifuge tube. The tube was then cooled at 4 °C for 20 min and shaken up and down to ensure complete extraction. The filtered solution was subsequently analyzed using a spectrophotometer to measure the absorbance at 280 nm and 325 nm. The total phenolic content was expressed as OD_280 nm_ per gram of fresh weight, while the flavonoid content was represented as OD_325 nm_ per gram of fresh weight.

### 2.11 Effects of bamboo vinegar, *C. laurentii*, and their combined treatment on the storage quality of grapefruit

The grapefruits underwent treatment following the method outlined in Section 2.4. On the 28th day after treatment, samples were collected and ground into powder while submerged in liquid nitrogen. To evaluate the postharvest nutritional quality of the grapefruits, the contents of total soluble solids (TSS), ascorbic acid (AsA), titratable acidity (TA) and reducing sugars (RS) were measured following the methods of Liu et al. (2011) and Wu et al. (2017). Each treatment was assessed three times, and the entire experiment was conducted two additional times. The grapefruits' weight was measured on days 0, 7, 14, 21, and 28 using an electronic balance accurate to 0.01 g, following the method of Guan et al. (2025). The calculation method for weight loss rate is as follows: Weight loss rate (%) = [(Initial fruit weight - Final fruit weight) / Initial fruit weight] × 100. Each treatment underwent six measurements, and the experiment was carried out two additional times. The color characteristics of the fruits (L*, a*, b*, c*, and *h*^*0*^) were assessed using a colorimeter, following the approach of Kozakiewicz et al. (2025). The L* value represents brightness, ranging from 0 to 100; the a* value indicates the degree of green (−120) to red (+120); and the b* value represents the degree of blue (−120) to yellow (+120). Chroma (c*) was calculated using the following equation:

(1) $$c^{*}=\sqrt{a^{*2}+b^{*2}}$$

Hue angle (*h*^*0*^) was calculated using the following equation:

(2) $$\begin{array}{c} h^{0}=arctan\left(b^{*}/a^{*}\right) \end{array}$$

For each sample, color measurements were taken ten times, and the experiment was repeated twice.

Given that the main objective of this study is to evaluate the final impact of the treatment on the post-storage quality of grapefruit, and considering that previous studies have confirmed that the trends of TSS, TA, AsA and RS during storage are relatively stable, these nutritional quality indicators were measured on the 0th day (initial value) and the 28th day (storage end). While indicators such as weight loss rate and color parameters, which are more sensitive to physiological changes, were monitored dynamically every 7 days to comprehensively reflect the continuous impact of the treatment on the fruit quality.

### 2.12 Statistical analysis

A completely randomized design was implemented for all experimental trials. Data were analyzed using Duncan's multiple comparison test in SPSS version 20.0, with statistical significance set at *P* < 0.05. Experimental datasets were systematically organized using Microsoft Excel 2020, while graphical representations were created with OriginPro 2020.

## 3 Results

### 3.1 Preliminary screening of antifungal activity of different concentrations of *C. laurentii* against *Penicillium*

Fig. 1A shows that *C. laurentii* at 10^7^ CFU mL^−1^ consistently demonstrated the strongest inhibition of *P. digitatum* spore germination across all time points, with significantly reduced rates compared to the control. Specifically, the spore germination rates in the CK at 10 h, 12.5 h, and 19 h were 9.25%, 36.90% and 63.97%, respectively. These rates were 31.52%, 91.85% and 41.00% higher than those observed in the 10^7^ CFU mL^−1^*C. laurentii* group at the corresponding time points. Fig. 1B indicates that no significant differences were observed among the treatment groups at 10 h. However, at 12.5 h and 19 h, the germ tube lengths in the groups treated with 10^6^ and 10^7^ CFU mL^−1^*C. laurentii* were significantly shorter than those in the control group. Notably, the germ tube length in the group treated with 10^7^ CFU mL^−1^*C. laurentii* was the shortest, being only 0.75- and 0.94-fold that of the control group at 12.5 h and 19 h, respectively. Fig. 1C illustrates that treatment with 1 × 10^7^ CFU mL^−1^ of *C. laurentii* significantly reduced the spore germination rate of *P. italicum* at 9 h compared to the control group, with the germination rate in the treated group being only 0.62-fold that of the control. Notably, no significant differences in spore germination rates were observed between the control and the 1 × 10^7^ CFU mL^−1^*C. laurentii*-treated groups at 7 h and 8 h. As for germ tube length, Fig. 1D shows that at 7 h, there were no notable differences in the germ tube lengths of *P. italicum* between the CK and those treated with 1 × 10^5^, 1 × 10^6^, or 1 × 10^7^ CFU mL^−1^ of *C. laurentii*. However, at 8 h post-inoculation, the germ tube lengths in the groups treated with 1 × 10^5^, 1 × 10^6^, and 1 × 10^7^ CFU mL^−1^ of *C. laurentii* were significantly shorter than those in the CK, showing reductions of 7.09%, 14.88% and 12.63%, respectively. Importantly, at 9 h post-inoculation, only the group treated with 1 × 10^7^ CFU mL^−1^ of *C. laurentii* exhibited a significantly shorter germ tube length compared to the control, with a reduction of 12.55%. Fig. 1E demonstrates that at 6 h, 7 h and 9 h post-inoculation, the spore germination rates of *P. expansum* in the CK were notably higher than those in the groups treated with 1 × 10^5^, 1 × 10^6^, and 1 × 10^7^ CFU mL^−1^ of *C. laurentii*. Notably, at 9 h post-inoculation, the spore germination rate of *P. expansum* in the CK reached its peak at 79.97%, which was 1.42 times that observed in the group treated with 1 × 10^7^ CFU mL^−1^ of *C. laurentii*. As for germ tube length, Fig. 1F reveals that only at 6 h post-inoculation did the treatment with 1 × 10^7^ CFU mL^−1^ of *C. laurentii* result in a significantly shorter germ tube length of *P. italicum* compared to the CK, with the treated group's length being 0.90-fold that of the CK.

**Fig. 1:**
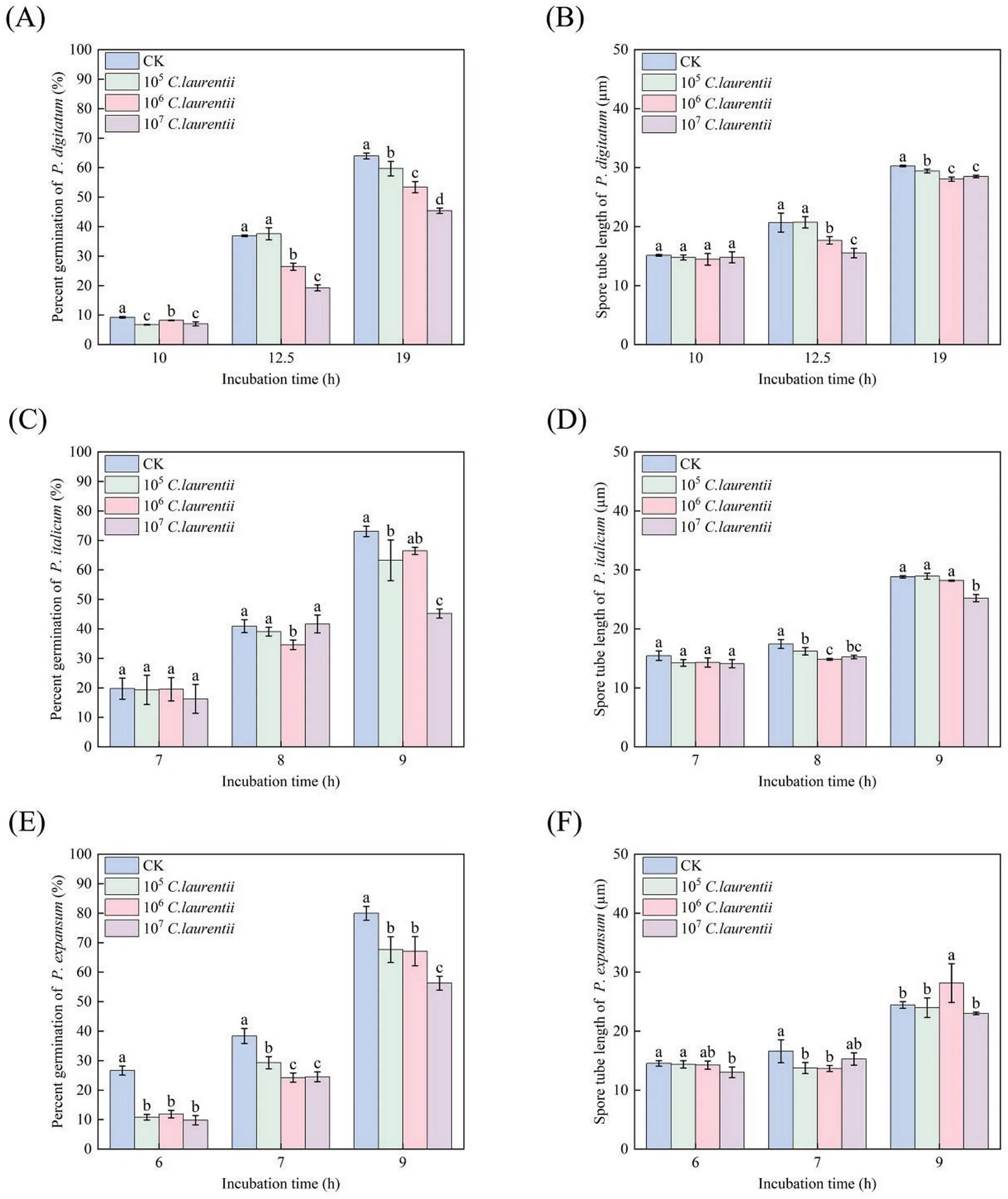
Effects of different concentrations of *C. laurentii* on the spore germination rate and germ tube length of *Penicillium*. (A), (C), and (E) illustrate the spore germination rates of *P. digitatum*, *P. italicum,* and *P. expansum*, respectively, under treatment with varying concentrations of *C. laurentii*. (B), (D), and (F) depict the germ tube lengths of *P. digitatum*, *P. italicum*, and *P. expansum*, respectively, under the same treatment conditions.

Treatments showing statistically significant differences are denoted by different letters (Duncan's test, *P* < 0.05).

### 3.2 Preliminary screening of antifungal activity of different concentrations of bamboo vinegar against *Penicillium*

Fig. 2A demonstrates that the spore germination rate of *P. digitatum* decreased with increasing concentrations of bamboo vinegar at all observed time - points. at 19 h post-inoculation, the spore germination rate of *P. digitatum* in the CK was significantly higher than those in the 0.1%, 0.5%, and 1.0% bamboo vinegar treatment groups. Specifically, it was 1.86-, 2.65-, and 7.10-fold the rates of the 0.1%, 0.5%, and 1.0% bamboo vinegar treatment groups, respectively. Fig. 2B shows that the germ tube length of *P. digitatum* in all treatment groups gradually increased over time, peaking at 19 h post-inoculation. At this time, the germ tube length of *P. digitatum* in the 1% bamboo vinegar treatment group was significantly shorter than that in the CK, being 37.49% less than the control. Fig. 2C demonstrates that at all observed time intervals, the spore germination rate of *P. italicum* in the CK was significantly higher than that in the group treated with 1.0% bamboo vinegar. The germination rates of *P. italicum* in the control, 0.1%, 0.5% and 1.0% BV treatment groups peaked at 9 h post-inoculation, reaching 93.27%, 96.25%, 94.43%, and 36.74%, respectively. Fig. 2D shows that at all observed time points, the germ tube lengths of *P. italicum* in the 0.5% and 1.0% BV treatment groups were notably shorter compared to the CK. Specifically, at 8 h post-inoculation, the germ tube lengths in the 0.5% and 1.0% BV treatment groups were 0.78- and 0.70-fold those of the CK, respectively. At 9 h, the germ tube lengths in the 0.5% and 1.0% BV treatment groups were reduced by 42.77% and 55.83%, respectively, compared to the control group. Fig. 2E reveals that, across all observed time intervals, the spore germination rates of *P. expansum* in the 0.5% and 1.0% bamboo vinegar treatment groups were lower than those in the CK, significantly. At 9 h post-inoculation, the germination rate of *P. expansum* in the CK exhibited a marked increase compared to the groups treated with 0.1%, 0.5%, and 1.0% BV, registering at 1.22 times, 1.41 times, and 2.56 times the rates observed in the corresponding BV-treated groups, respectively. At each time point, the germ tube lengths of *P. expansum* in the 0.5% and 1.0% BV treatment groups were significantly shorter than those in the CK. The germ tube lengths of *P. expansum* in all treatment groups reached their maximum at 9 h. At this time point, the germ tube lengths in the 0.1%, 0.5%, and 1.0% BV treatment groups were shorter than that in the CK, being 0.70-, 0.67-, and 0.48-fold the length of the CK, respectively (Fig. 2F).

**Fig. 2:**
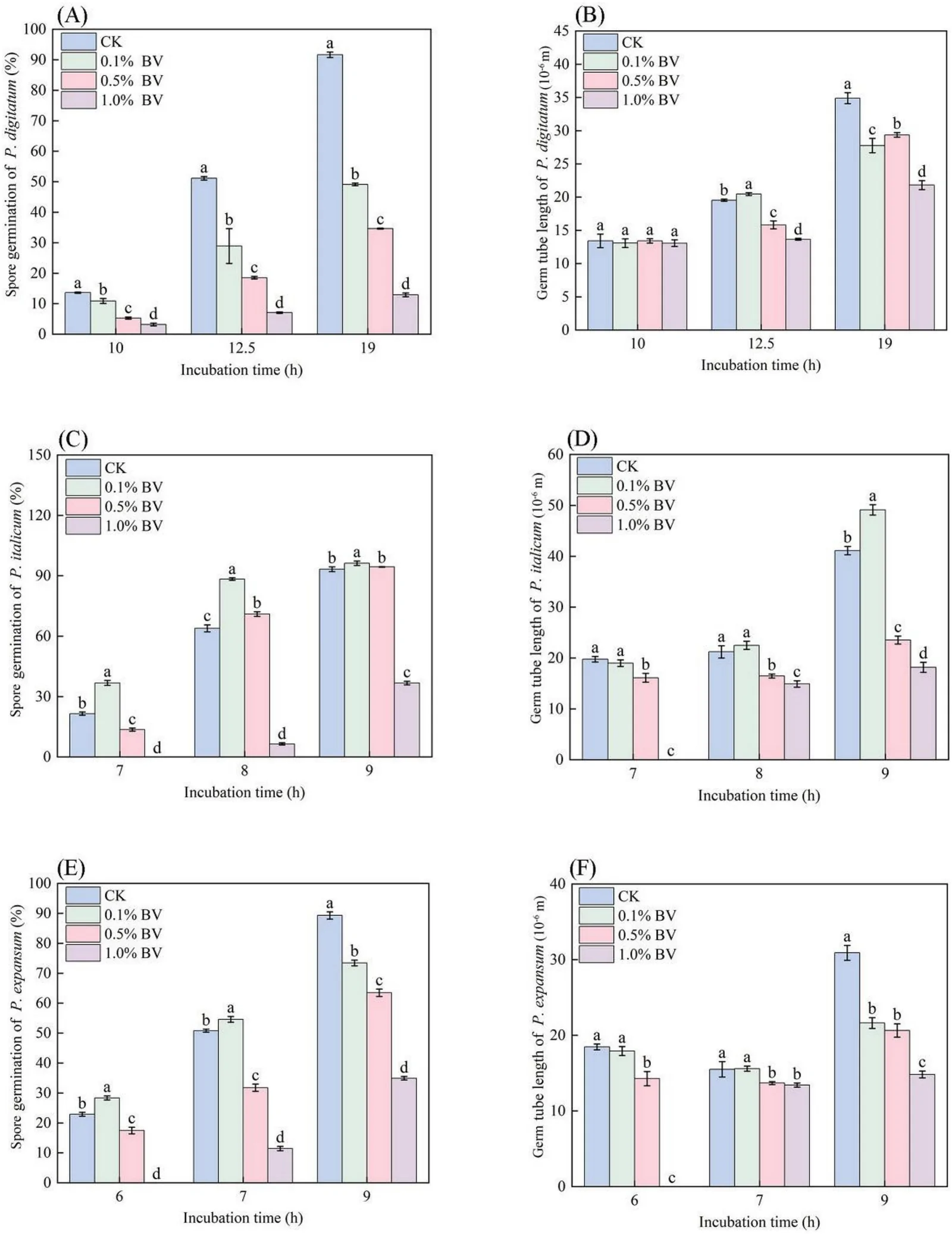
Effects of different concentrations of bamboo vinegar on the spore germination rate and germ tube length of *Penicillium*. (A), (C), and (E) illustrate the spore germination rates of *P. digitatum*, *P. italicum,* and *P. expansum*, respectively, under treatment with varying concentrations of bamboo vinegar. (B), (D), and (F) depict the germ tube lengths of *P. digitatum*, *P. italicum*, and *P. expansum*, respectively, under the same treatment conditions.

Treatments marked with different letters exhibit statistically significant differences (Duncan's test, *P* < 0.05).

### 3.3 Impact of various bamboo vinegar concentrations on *C. laurentii* growth

Fig. 3 illustrates the trends in the viable count of *C. laurentii* over different culture durations (6, 12, 24, and 48 h) under varying concentrations of bamboo vinegar. As the culture time progressed, the viable count of *C. laurentii* increased gradually across all BV concentrations. Overall, no significant inhibitory effect on *C. laurentii* growth was observed with different bamboo vinegar concentrations throughout the culture period. Notably, after 24 h of incubation, 0.2% and 1% BV exhibited a significant stimulatory effect on *C. laurentii* growth. Given that, as demonstrated in Section 3.2, 1% bamboo vinegar demonstrated the most effective inhibition on the spore germination rate and germ tube elongation of *P. digitatum*, *P. italicum*, and *P. expansum*, 1% BV was selected for subsequent experiments.

**Fig. 3:**
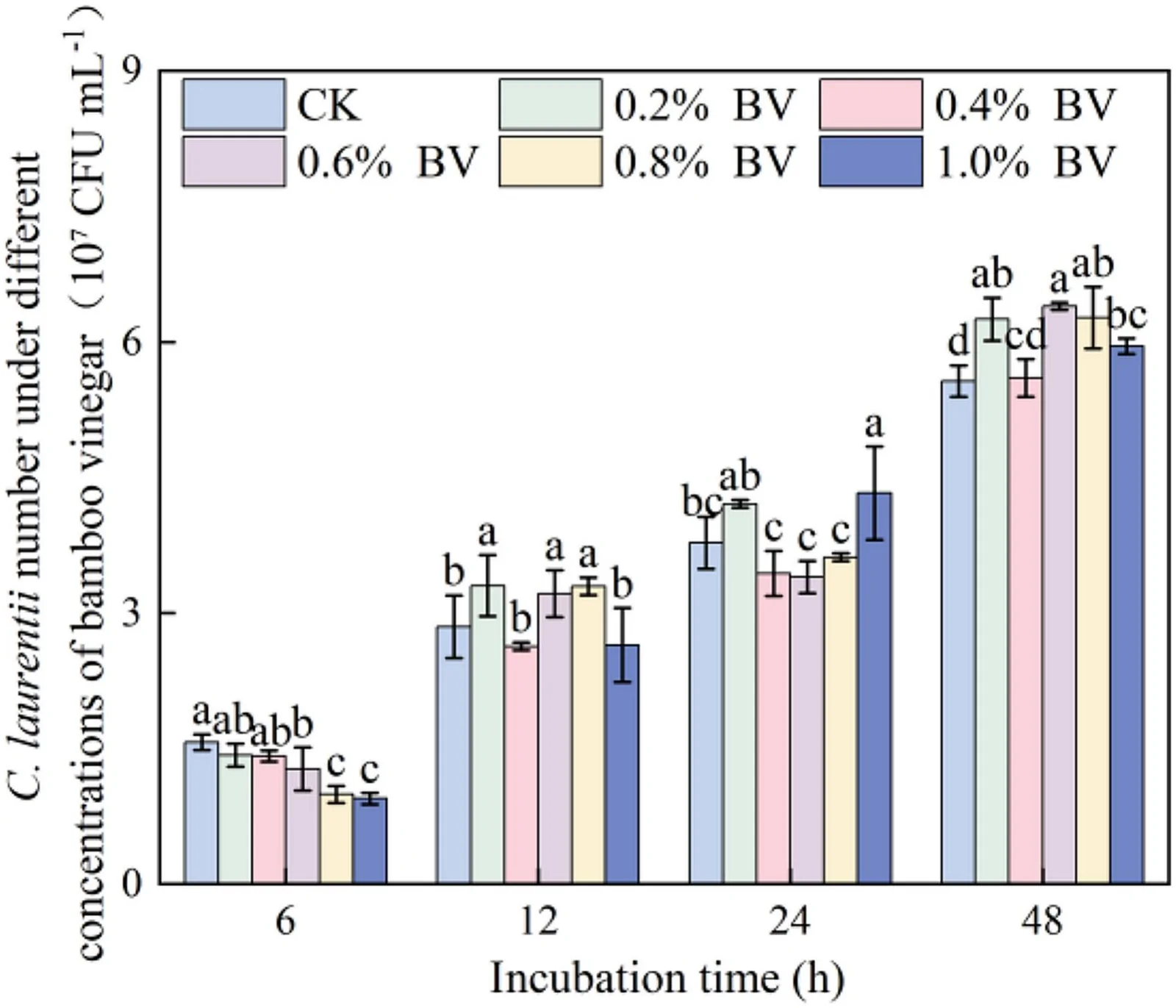
Impact of various bamboo vinegar concentrations on *C. laurentii* growth.

Different letters indicate significant differences (Duncan's test, *P* < 0.05).

### 3.4 Impact of bamboo vinegar-induced *C. laurentii* on the spore germination rate and tube length of *Penicillium*

Fig. 4A and B demonstrate that, in comparison to the group treated solely with *C. laurentii*, the BV-*C. laurentii* combination treatment significantly suppressed both the spore germination rate and germ tube length of *P. digitatum*. At 19 h, the spore germination rate in the *C. laurentii* treatment group reached its peak at 75.57%, whereas in the BV-*C. laurentii* treatment group, it was only 14.43%. Additionally, the tube length in the BV-*C. laurentii* treatment group was 2.06 × 10^−5^ m, which was 0.72 times that of the *C. laurentii* treatment group. Across all observed time points, the BV-*C. laurentii* treatment group consistently exhibited a notable inhibitory effect on both the spore germination rate and germ tube length of *P. italicum* when compared to the group treated solely with *C. laurentii*. At 9 h, the spore germination rate in the BV-*C. laurentii* treatment group was merely 0.08 times that of the *C. laurentii* treatment group (Fig. 4C). Furthermore, at the same time point, the tube length of *P. italicum* in the *C. laurentii* group was 6.62% longer than that in the BV-*C. laurentii* treatment group (Fig. 4D). Fig. 4E and F illustrate that both the spore germination rate and germ tube length of *P. expansum* were significantly greater in the *C. laurentii* group than in the BV-*C. laurentii* group at 9 h, both *C. laurentii* and BV-*C. laurentii* treatment groups reached their peak spore germination rates, with values of 74.57% and 13.43%, respectively. Concurrently, the tube lengths were 2.20 × 10^−5^ m in *C. laurentii* treatment group and 1.68 × 10^−5^ m in BV-*C. laurentii* treatment group.

**Fig. 4:**
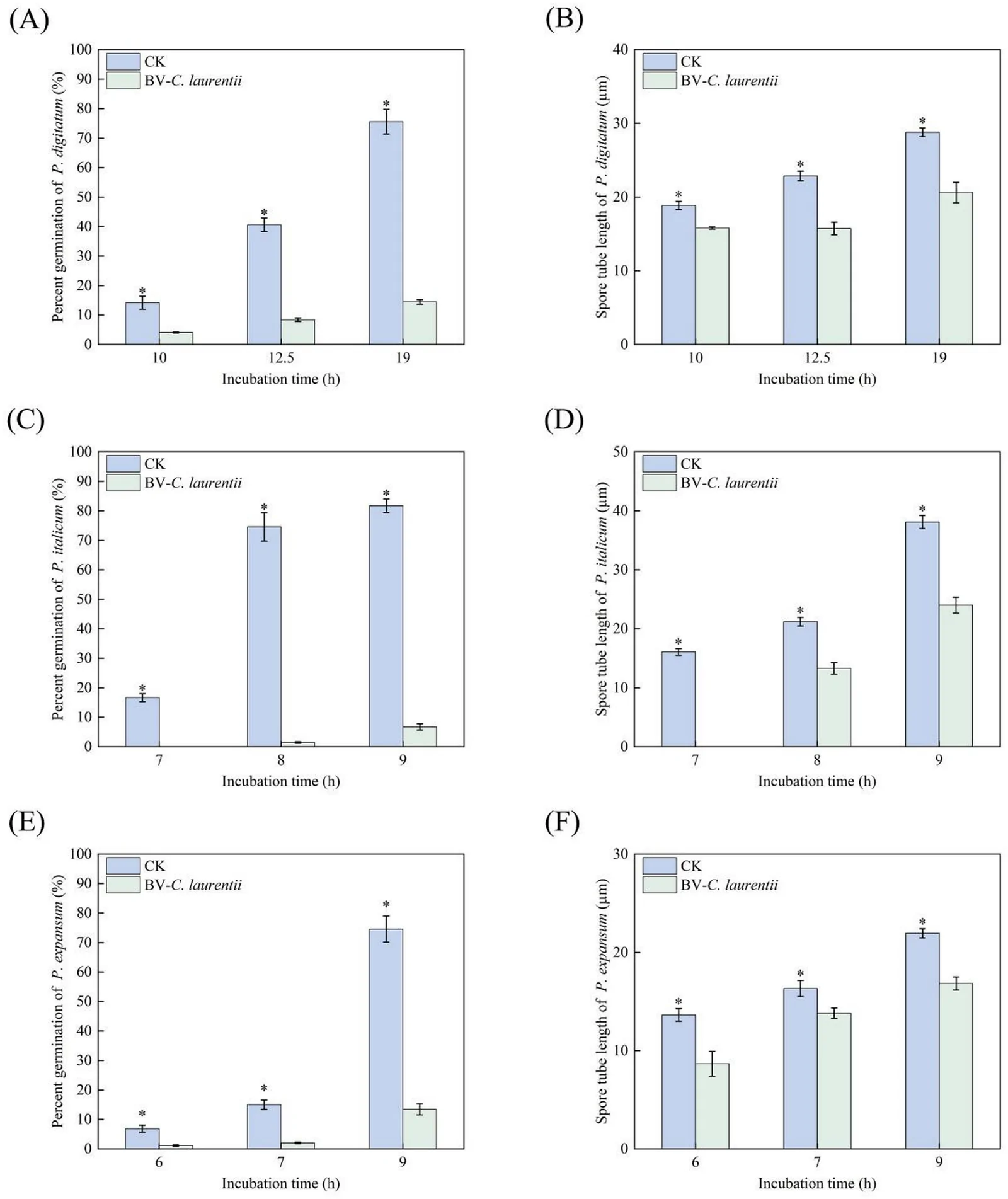
Inhibitory effects of bamboo vinegar-induced *C. laurentii* on the spore germination rate and germ tube length of *Penicillium*. (A), (C), and (E) illustrate the spore germination rates of *P. digitatum*, *P. italicum*, and *P. expansum*, respectively, following treatment with BV-*C. laurentii* and *C. laurentii*. (B), (D), and (F) depict the corresponding germ tube lengths of *P. digitatum*, *P. italicum*, and *P. expansum* following treatment with BV-*C. laurentii* and *C. laurentii*.

Data are shown as mean ± SE. Asterisks indicate significant differences (*P* < 0.05, *t*-test) between the *C. laurentii* and BV-*C. laurentii* groups at the indicated time points.

### 3.5 Imapct of BV-*C. laurentii* on cell wall integrity and cell membrane permeability in *P. digitatum*

The results of CFW are illustrated in Fig. 5A and C. In the BV-*C. laurentii* group, the blue fluorescence intensity of *P. digitatum* exhibited a declining trend over time. At every observed time point, the blue fluorescence levels of *P. digitatum* in the BV-*C. laurentii* treatment group were markedly lower compared to those in the corresponding control groups. Notably, at 48 h, the blue fluorescence value of *P. digitatum* in the BV-*C. laurentii* treatment group reached its lowest level, being only 0.42 times that of the control group. Treatment with BV-*C. laurentii* induced a marked increase in the red fluorescence intensity of *P. digitatum* relative to the control group across all time points (Fig. 5B, D). Specifically, at 48 h, the red fluorescence value of *P. digitatum* in the BV-*C. laurentii* treatment group peaked, reaching 3.18 times that of the control group. These findings suggest that during the experimental period, treatment with BV-*C. laurentii* may compromise the integrity of the cell wall and cell membrane of *P. digitatum* hyphae.

**Fig. 5:**
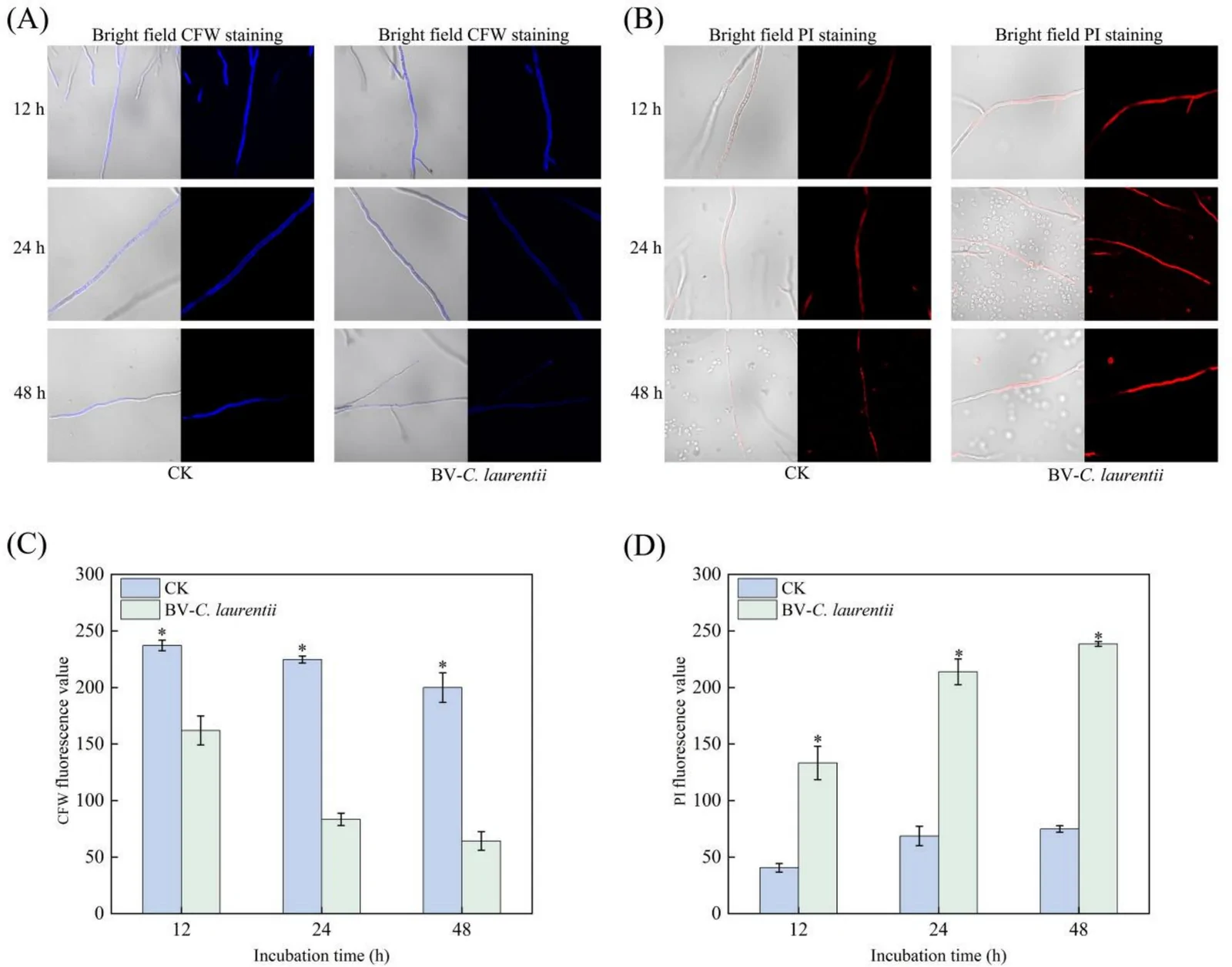
Fluorescence microscopy analysis of cell wall integrity and membrane permeability in *Penicillium digitatum* hyphae stained with CFW and PI. CFW (A). PI (B). Fluorescence value of the CFW staining (C). Fluorescence value of the PI staining (D). Bar = 50.00 μm (A-B). Asterisks show that there are significant differences (*P* < 0.05) between CK and BV-*C. laurentii* treatment groups at the shown time points, based on *t*-test.

### 3.6 Tolerance of *C. laurentii* induced by bamboo vinegar to oxidative stress under different concentrations of H₂O₂

Fig. 6 illustrates that as the concentration of H₂O₂ increased, the viable cell count of *C. laurentii* exhibited a decreasing trend. When the H₂O₂ concentration was 0%, the viable cell count of the treatment group BV-*C. laurentii* was significantly lower than that of the non-induced *C. laurentii* group, with a reduction of 33.33% compared to the viable cell count of the *C. laurentii* treatment group. At an H₂O₂ concentration of 5%, both the *C. laurentii* and BV-*C. laurentii* treatment groups reached their lowest viable cell counts, which were 5.47 × 10^6^ CFU mL^−1^ and 3.67 × 10^6^ CFU mL^−1^, respectively. From an overall perspective, as the H₂O₂ concentration rose, the decline in the viable cell count of the BV-*C. laurentii* treatment group was less pronounced than that of the *C. laurentii* treatment group. This observation indicates that bamboo vinegar induction can enhance the tolerance of *C. laurentii* to oxidative stress.

**Fig. 6:**
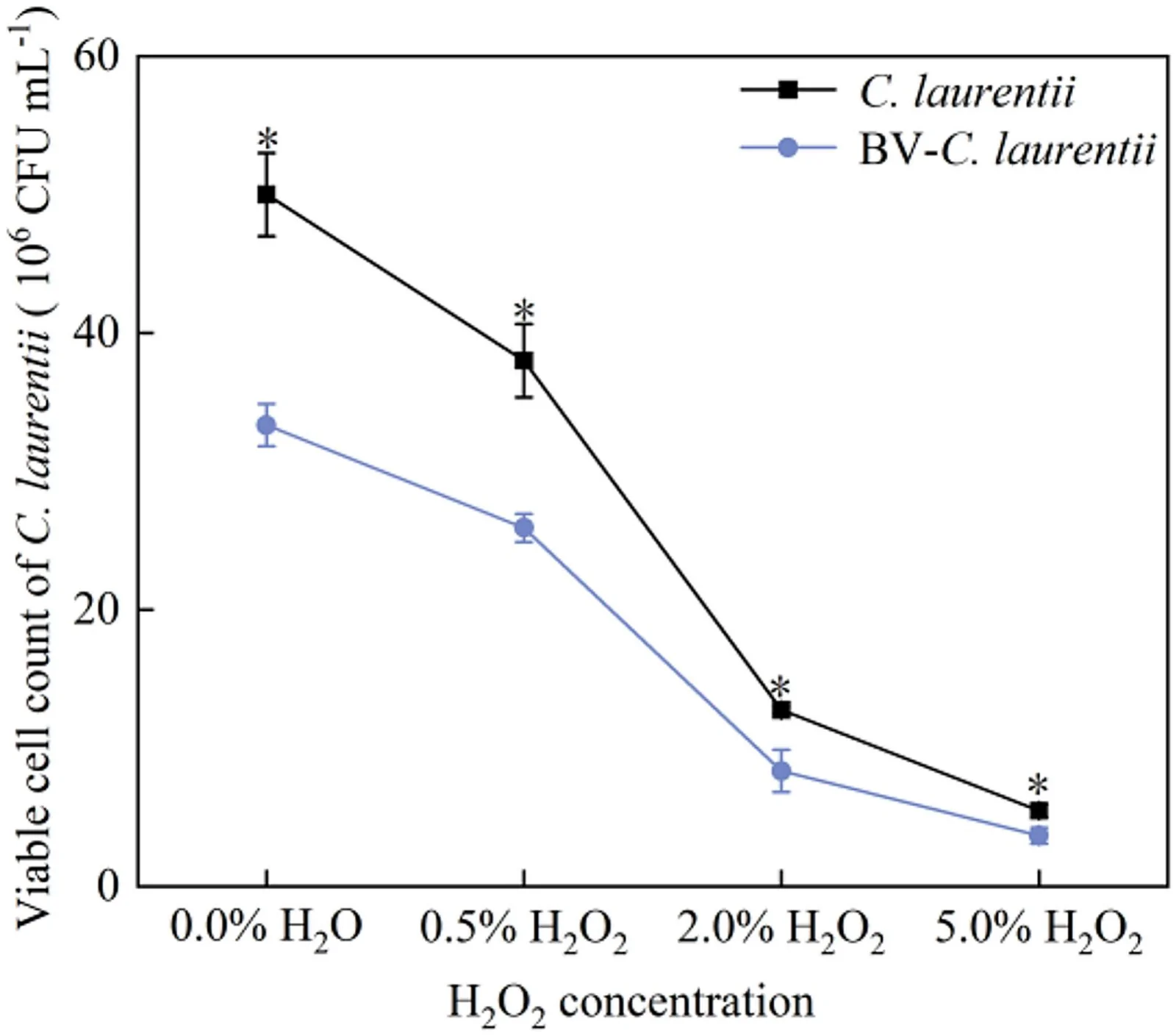
Viable cell counts of *C. laurentii* and BV-*C. laurentii* in the treatment group under different concentrations of H₂O₂ Data represent mean ± standard error (SE). At the specified time points, asterisks denote statistically significant differences (*P* < 0.05) between the *C. laurentii* and BV-*C. laurentii* treatment groups, as determined by *t*-test.

### 3.7 Determination of oxidative stress-related indicators and defensive enzyme activities in *C. laurentii* induced by bamboo vinegar

As shown in Table 1, there was no significant difference in CAT activity between the treatment groups of *C. laurentii* and BV-*C. laurentii*. MDA, the final product of membrane lipid peroxidation, serves as a direct indicator of cell membrane damage severity. Following 48 h of cultivation, the MDA level in the BV-*C. laurentii* treatment group was significantly lower than that in the *C. laurentii* treatment group, specifically 0.87 - fold of the MDA content in the *C. laurentii* group. GSH content and GR activity, as core components of the antioxidant defense system, work together to maintain the intracellular redox balance and protect cells from oxidative damage. After 48 h of cultivation, the GSH content in the BV-*C. laurentii* treatment group was significantly lower than that in the *C. laurentii* treatment group, reaching 1.4 × 10^−4^ mol L^−1^. Meanwhile, the GR activity in the BV-*C. laurentii* treatment group after 48 h of cultivation was significantly higher than that in the *C. laurentii* treatment group, at 5.77 U L^−1^. Table 1 also indicates that both *C. laurentii* and BV-*C. laurentii* possess the ability to produce CHI and GLU. Notably, the CHI and GLU activities in the BV - *C. laurentii* treatment group were significantly higher than those in the *C. laurentii* group, being 1.05 times and 1.22 times higher, respectively.

**Table 1:** The values of oxidative stress - related indicators and defensive enzyme activities in *C. laurentii* induced by bamboo vinegar.

Oxidative stress-related indicators and defensive enzyme	Treatment	Enzyme activity or substance content
CAT (U L^−1^)	*C. laurentii*	23.01 ± 1.02
	BV-*C. laurentii*	22.37 ± 2.46
MDA (μmol L^−1^)	*C. laurentii*	2.27 ± 0.01*
	BV-*C. laurentii*	1.98 ± 0.00
GSH (10^−3^ mol L^−1^)	*C. laurentii*	0.16 ± 0.01*
	BV-*C. laurentii*	0.14 ± 0.01
GR (U L^−1^)	*C. laurentii*	2.39 ± 0.25
	BV-*C. laurentii*	5.77 ± 0.17*
CHI (10^3^ U L^−1^)	*C. laurentii*	1.09 ± 0.01
	BV-*C. laurentii*	1.14 ± 0.00*
GLU (10^3^ U L^−1^)	*C. laurentii*	0.48 ± 0.01
	BV-*C. laurentii*	0.58 ± 0.00*

Data represent mean ± standard error (SE). An asterisk indicates significant differences (*P* < 0.05) in oxidative stress markers or defense enzymes between the *C. laurentii* group and the BV-*C. laurentii* group, as determined by *t*-tests.

### 3.8 Inhibitory effects of bamboo vinegar, *C. laurentii*, and their individual and combined treatments on grapefruit green mold disease

In Table 2, the incidence rates of *P. digitatum*, *P. italicum*, and *P. expansum* on grapefruits are presented. Overall, as the storage time increased, the incidence rates in all treatment groups showed an upward trend. For *P. digitatum*, no significant differences in disease incidence were detected among treatment groups on days 4 and 5 of storage. By day 6, however, the control group exhibited a 100% infection rate, which was significantly higher than that observed in all treated fruit. Fig. 7A illustrates that the lesion diameter of grapefruit fruits increased as storage time extended. Compared with the CK, the treatment groups BV, *C. laurentii*, and BV-*C. laurentii* all significantly inhibited the spread of *P. digitatum* on grapefruits. Notably, the treatment group BV-*C. laurentii* demonstrated a significantly superior inhibitory effect on the spread of *P. digitatum* on grapefruit compared with the BV and *C. laurentii* treatment groups from day 5 to day 20. In Table 2, during day 4 to day 6 of storage, for *P. italicum* on grapefruits, only the *C. laurentii* group showed a significantly lower incidence rate than the CK. As shown in Fig. 7B, compared with the CK, the treatment groups BV, *C. laurentii*, and BV-*C. laurentii* all significantly inhibited the spread of *P. italicum* on grapefruits. On day 20, the lesion diameters of grapefruits in the CK, *C. laurentii*, BV, and BV-*C. laurentii* treatment groups reached their maximum values, measuring 5.21 cm, 3.79 cm, 3.65 cm, and 4.39 cm, respectively. In Table 2, during day 4 to day 6 of storage, for *P. expansum* on grapefruits, only the treatment group *C. laurentii* had a significantly lower incidence rate than the CK. As shown in Fig. 7C, only the *C. laurentii* treatment group displayed a significantly smaller lesion diameter than the control throughout storage. The lesion diameter reached its maximum value on day 30 of storage. Specifically, the lesion diameters in the *C. laurentii*, BV, and BV - *C. laurentii* treatment groups were 0.29, 1.29, and 1.21 time that of the control, respectively.

**Table 2:** Effects of different treatments on the incidence rate of diseases caused by *P. digitatum*, *P. italicum* and *P. expansum* in grapefruit.

Storage days (d)	Treatment	*P. digitatum* (%)	*P. italicum* (%)	*P. expansum* (%)
4	CK	58.33 ± 14.43 a	91.67 ± 14.43 a	83.33 ± 14.43 a
	*C. laurentii*	33.33 ± 14.43 a	41.68 ± 14.43 b	25.00 ± 0.00 b
	BV	41.67 ± 14.43 a	100.00 ± 0.00 a	75.00 ± 0.00 a
	BV-*C. laurentii*	50.00 ± 0.00 a	100.00 ± 0.00 a	66.67 ± 14.43 a
5	CK	58.33 ± 14.43 a	100.00 ± 0.00 a	91.67 ± 14.43 a
	*C. laurentii*	50.00 ± 0.00 a	58.33 ± 14.43 b	50.00 ± 25.00 b
	BV	50.00 ± 0.00 a	100.00 ± 0.00 a	100.00 ± 0.00 a
	BV-*C. laurentii*	58.33 ± 14.43 a	100.00 ± 0.00 a	91.67 ± 14.43 a
6	CK	100.00 ± 0.00 a	100.00 ± 0.00 a	100.00 ± 0.00 a
	*C. laurentii*	50.00 ± 0.00 b	58.33 ± 14.43 b	50.00 ± 0.00 b
	BV	66.67 ± 14.43 b	100.00 ± 0.00 a	100.00 ± 0.00 a
	BV-*C. laurentii*	66.67 ± 14.43 b	100.00 ± 0.00 a	100.00 ± 0.00 a

Statistically significant differences among treatments are indicated by distinct letters (Duncan's test, *P* < 0.05).

**Fig. 7:**
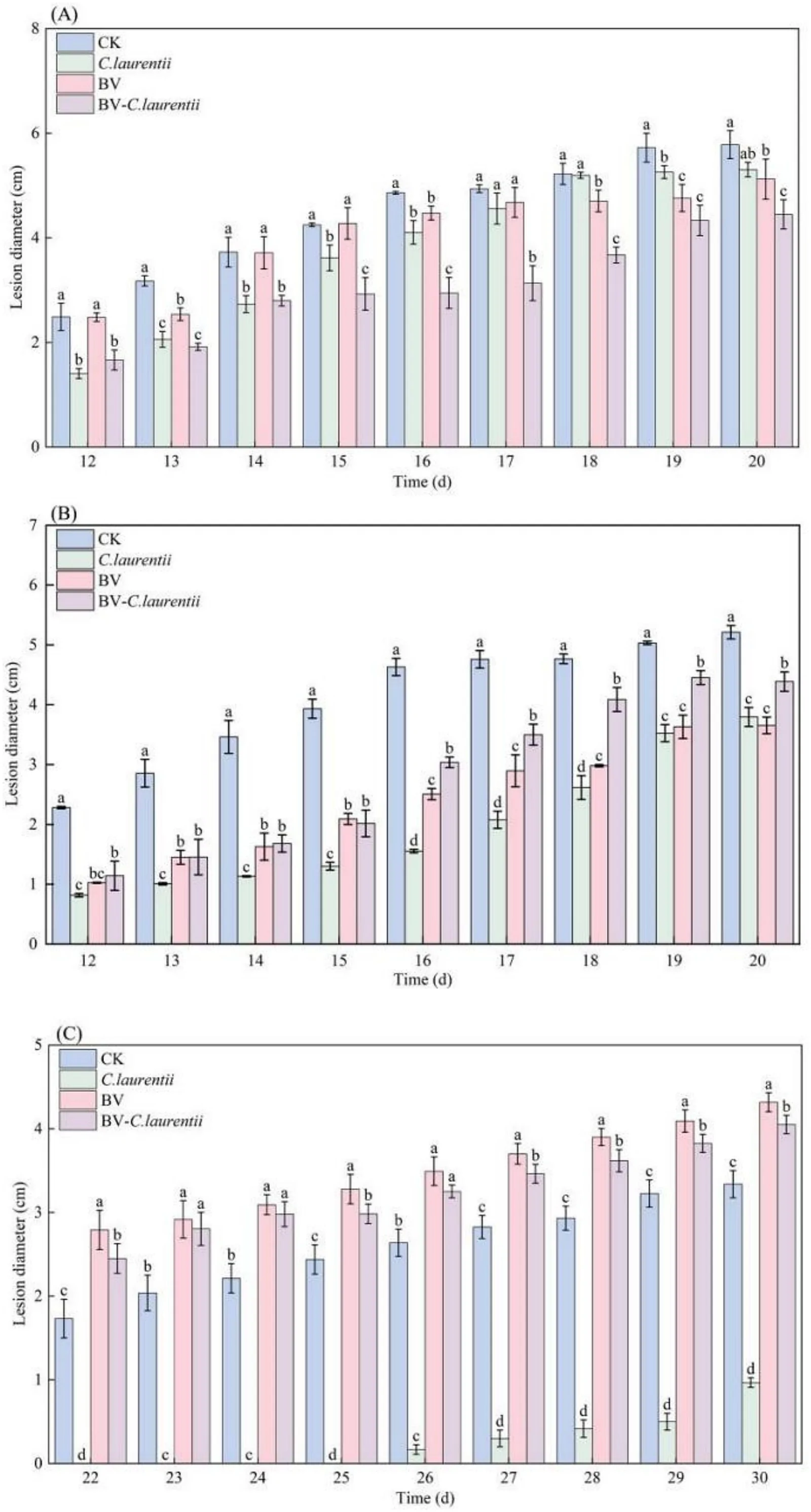
Effect of different treatments on the diameter of disease spots caused by *P. digitatum* (A), *P. italicum* (B), and *P. expansum* (C) in grapefruit The data are expressed as mean ± standard error (SE). Significant differences among treatments (Duncan's test, *P* < 0.05) are indicated by different letters.

### 3.9 Effects of individual and combined treatments of bamboo vinegar and *C. laurentii* on defense-related compounds and enzyme activity in grapefruit

Fig. 8A demonstrates that CHI activity in grapefruit initially increased during early storage, reaching a peak at 14 days post-treatment. The CHI activity in treatment groups treated with *C. laurentii*, BV, and BV-*C. laurentii* was 1.36-, 1.32-, and 1.30-fold higher than that of the CK, respectively. Fig. 8B reveals that, across the entire storage duration, GLU activity in the *C. laurentii*, BV, and BV-*C. laurentii* treatment groups was significantly elevated compared to the control. Notably, the GLU activity in the BV-*C. laurentii* treatment groups reached its maximum on day 14, with values being 5.74, 1.18, and 2.32 times that of the CK, *C. laurentii* and BV treatment group, respectively. From the 7th day to the 28th day of storage, both the total phenolic content and flavonoid content in the BV-*C. laurentii* group were significantly higher than those in the CK. The total phenolic content peaked on the 7th day, with the BV-*C. laurentii* treatment exhibiting a 19.28% increase compared to the CK. Meanwhile, the flavonoid content reached its maximum on the 28th day, where the BV-*C. laurentii* treatment showed a 1.20-fold elevation relative to the CK (Fig. 8C and D). Fig. 8E shows that from day 7 to day 28, PAL activity in both the *C. laurentii* and BV-*C. laurentii* treatment groups was significantly elevated compared to the CK. The PAL activity in the *C. laurentii* treatment group peaked at 93.67 U g^−1^ on day 28, while that in the BV-*C. laurentii* treatment group reached its maximum at 71.20 U g^−1^ on day 21. Fig. 8F indicates that PPO activity in the BV-*C. laurentii* treatment group was significantly higher than that in the control group from day 7 to day 28, with the highest activity of 31.65 U g^−1^ observed on day 21, which was 28.87% higher than that of the control group.

**Fig. 8:**
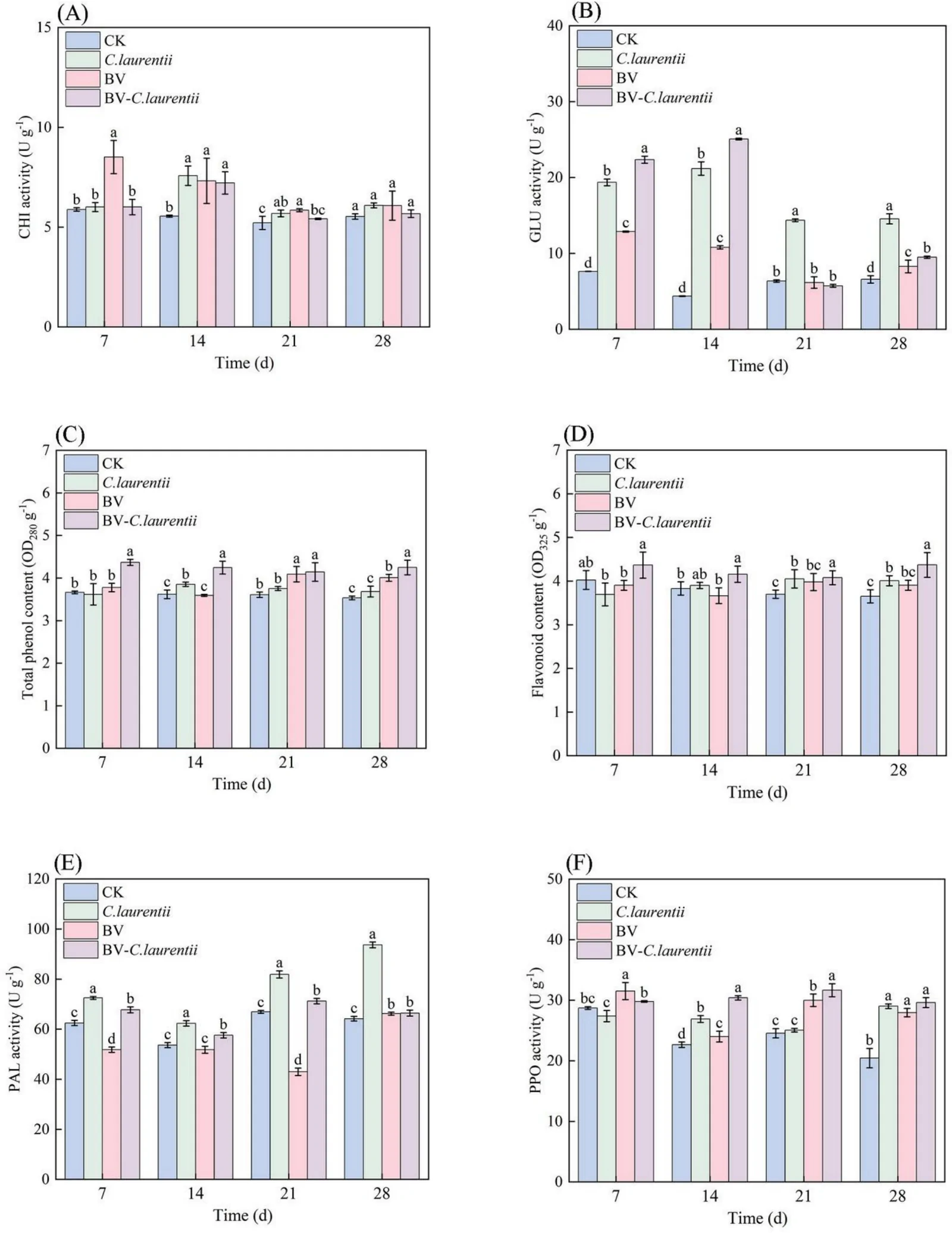
Effect of *C. laurentii* and bamboo vinegar treatment on the activity of CHI (A), GLU (B), PAL (E), PPO (F) and the content of total phenol (C), flavonoid (D) in grapefruit during storage Statistically significant differences between treatments are denoted by distinct letters, as determined by Duncan's multiple range test (*P* < 0.05). Data are presented as mean ± standard error (SE).

### 3.10 Effects of individual and combined treatments of bamboo vinegar and *C. laurentii* on the storage quality of grapefruit

#### 3.10.1 Impacts of individual and combined treatments of bamboo vinegar and *C. laurentii* on the nutritional quality indicators of grapefruit

Table 3 shows that on day 28, grapefruit treated with *C. laurentii* and BV-*C. laurentii* exhibited significantly higher titratable acid content compared to the CK. Compared with the CK, the titratable acid content increased by 10.42% after *C. laurentii* treatment and by 14.75% after BV-*C. laurentii* treatment. On day 28, grapefruit treated with *C. laurentii* and BV-*C. laurentii* exhibited significantly higher reducing sugar content than the control, with increases of 11.50% and 10.92%, respectively. For ascorbic acid content in grapefruit, *C. laurentii* and BV-*C. laurentii* treatments significantly increased levels compared to the control, reaching 1.34-fold and 1.17-fold of CK, respectively. In contrast, the BV treatment group showed no significant difference in ascorbic acid content compared to the CK. For soluble solids content, all three treatments (*C. laurentii*, BV, and BV-*C. laurentii*) significantly increased values relative to CK, with respective increases of 1.10-, 1.03-, and 1.15-fold.

**Table 3:** Effect of *C. laurentii* and bamboo vinegar treatment on fruit quality of grapefruit during storage.

Storage days(d)	Treatment	Soluble solid content (%)	Titratable acid (%)	Ascorbic acid (10^−2^ g kg^−1^)	Reducing sugar (%)
0	CK	11.03 ± 0.033	14.99 ± 0.101	6.60 ± 0.115	3.90 ± 0.050
28	CK	10.17 ± 0.033 d	13.79 ± 0.010 c	5.97 ± 0.057 c	3.48 ± 0.145 b
	*C. laurentii*	11.23 ± 0.033 b	14.04 ± 0.004 b	8.01 ± 0.119 a	3.86 ± 0.106 a
	BV	10.43 ± 0.033 c	13.15 ± 0.006 d	6.02 ± 0.033 c	3.56 ± 0.061 ab
	BV-*C. laurentii*	11.67 ± 0.033 a	14.28 ± 0.094 a	7.00 ± 0.144 b	3.88 ± 0.059 a

Significant differences in fruit nutritional quality among treatments at 28 d are indicated by different letters in the same column (Duncan's multiple range test, *P* < 0.05). Data are presented as mean ± SE.

#### 3.10.2 Effects of individual and combined treatments of bamboo vinegar and *C. laurentii* on the weight loss rate of grapefruit

As shown in Fig. 9A, with the increase in storage duration, the weight loss rate of grapefruit in all treatment groups exhibited an upward trend, reaching its maximum on day 28. Notably, on day 28, the weight loss rate of the BV-*C. laurentii* treatment group was significantly lower than that of both the control group and the *C. laurentii* treatment group, with reductions of 10.70% and 11.65%, respectively, compared to these two groups.

**Fig. 9:**
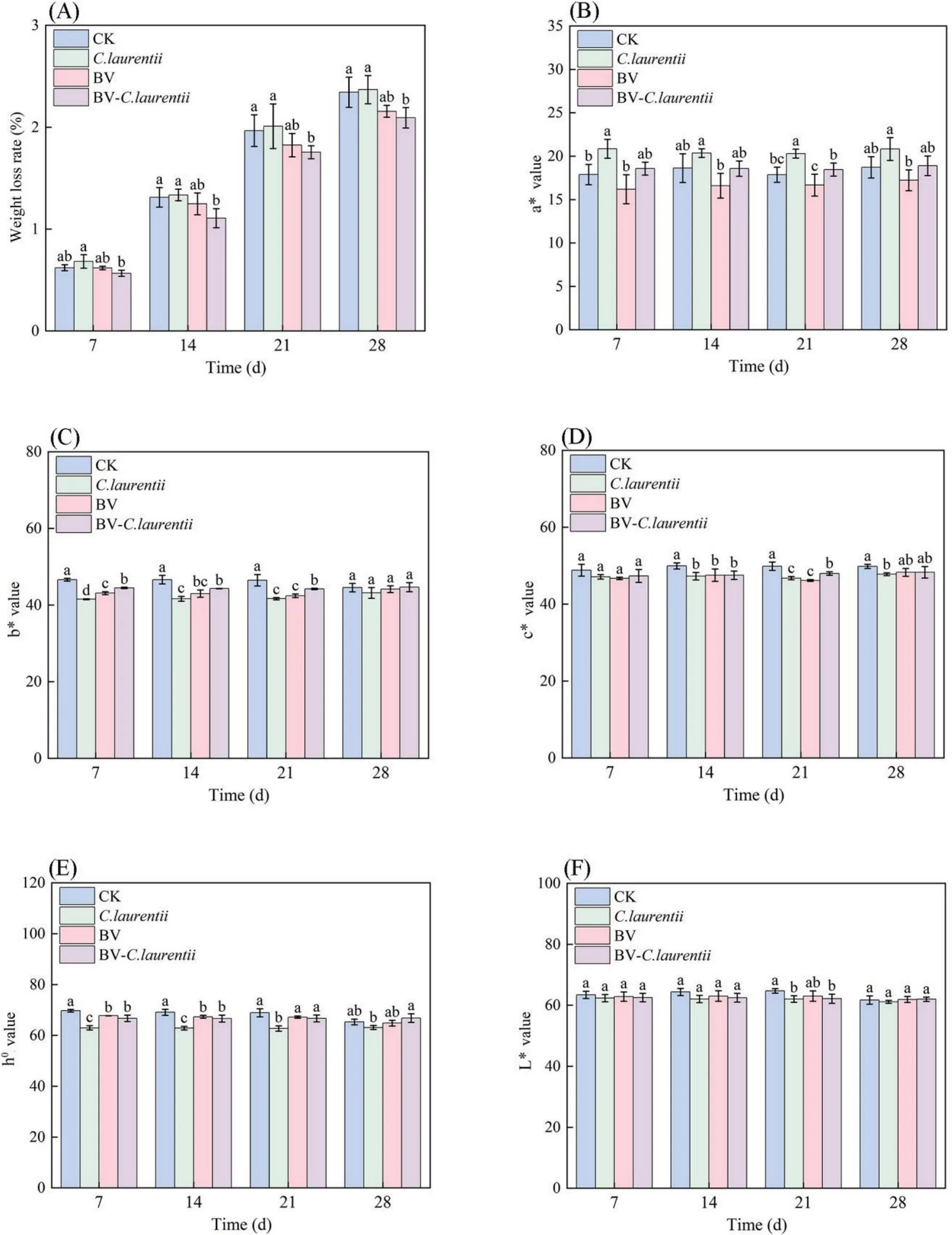
Effects of *C. laurentii* and bamboo vinegar treatment on weight and color of grapefruit during storage. (A) weight loss (B) a* value, (C) b* value, (D) c* value, (E) *h^0^* value, (F) L* value Treatments bearing different superscript letters exhibit statistically significant differences (*P* < 0.05), as evaluated through Duncan's multiple range test. All data are expressed as the mean ± standard error (SE).

#### 3.10.3 Effects of individual and combined treatments of bamboo vinegar and *C. laurentii* on the appearance quality of grapefruit

As depicted in Fig. 9B, throughout the entire storage period, the a* values of grapefruits in the treatment groups *C. laurentii*, BV, and BV-*C. laurentii* exhibited a gradual increase. Notably, the BV-*C. laurentii* treatment group exhibited no significant difference in the a* values compared to the control. Regarding b values. Fig. 9C reveals that while the CK and the BV-*C. laurentii* treatment group showed no significant changes over the storage period, the b* values in the *C. laurentii* and BV treatment groups increased slowly. Furthermore, during the period from day 7 to day 21, the control group exhibited significantly higher b* values compared to the *C. laurentii*, BV, and BV-*C. laurentii* treatment groups. Fig. 9D shows that there is no significant trend in c* values throughout the storage period. Fig. 9E indicates that from day 7 to day 28, the hue angle of the BV-*C. laurentii* treatment group remained almost constant, while that of the CK slowly decreased over the storage period. However, from day 21 to day 28, no significant differences in *h*^*0*^ values were detected among the control group, BV treatment group, and BV-*C. laurentii* treatment group. Finally, Fig. 9F illustrates that during the entire storage period, the L* values of grapefruits in the *C. laurentii*, BV, BV-*C. laurentii*, and control groups all gradually decreased. On day 21, the L* value of the CK was significantly higher than those of the *C. laurentii* and BV-*C. laurentii* treatment groups, whereas no significant differences in L* values were observed among the treatment groups at other time points.

## 4 Discussion

Grapefruit is highly susceptible to pathogen infection after harvest, which often leads to pulp softening, pitting, and decay, resulting in a substantial decline in its commercial value. Among the post - harvest diseases, green mold caused by *P. digitatum*, *P. italicum*, and *P. expansum* is one of the most destructive (Bhatta, 2022). Therefore, the development of efficient and safe post - harvest preservation techniques is crucial for delaying decay and maintaining the flavor and nutritional quality of grapefruit. Yeast, as a core microbial resource in biological control systems, offers several advantages, including strong antagonistic and stress - resistance properties, as well as being environmentally friendly (Pereyra et al., 2024). Our research team has conducted extensive experiments in the early stages, and the results demonstrate that *C. laurentii* can significantly suppress fungal pathogen-induced decay and quality deterioration in postharvest fruits (Deng et al., 2021; Wang et al., 2019; Wu et al., 2024; Yang et al., 2025; Zhou et al., 2025). Bamboo vinegar is primarily enriched with bioactive substances such as organic acids and phenols. It not only possesses the ability to directly inhibit the spore germination of pathogenic fungi but also demonstrates a relatively strong capacity for scavenging reactive oxygen species (ROS) (Wang et al., 2024). The synergistic use of natural plant extracts and antagonistic yeasts has emerged as a novel and green strategy for controlling post - harvest fruit decay (Zhang et al., 2020). Plant extracts, including bamboo vinegar, chitosan, and cinnamic acid, can rapidly suppress the spore germination of pathogenic fungi and eliminate ROS. In contrast, antagonistic yeasts further reinforce disease prevention effects through mechanisms such as site competition, secretion of hydrolytic enzymes, and induction of fruit resistance (Błaszczyk et al., 2022; Chen et al., 2025; Gu et al., 2021). The combined use of these two approaches not only significantly reduces the incidence of postharvest fruit diseases and maintains fruit quality but also effectively minimizes the utilization of chemical fungicides. Consequently, it offers a safe and sustainable solution for postharvest preservation of fruits and vegetables (Wu et al., 2025). For instance, when *Sporidiobolus pararoseus* Y16 was induced by glycine betaine, it significantly reduced the sporophore length and spore germination rate of *P. expansum*, effectively enhancing its biological control efficacy against blue mold decay in apples without any negative impacts on storage quality indicators (Abdelhai et al., 2019). Similarly, when *Meyerozyma guilliermondii* was treated with caffeic acid, its antioxidant system was activated, significantly improving its oxidative stress tolerance and thereby enhancing its control effectiveness against post - harvest pathogenic fungi *Botrytis cinerea* and *P. expansum* in kiwifruit (Li et al., 2023). In this study, the experimental results revealed that a combination of 1% bamboo vinegar and 10^7^ CFU mL^−1^ of *C. laurentii* exhibited the best inhibitory effect on the sporophore length and spore germination rate of *P. italicum*, *P. digitatum*, and *P. expansum*. Moreover, 1% bamboo vinegar did not inhibit the growth of 10^7^ CFU mL^−1^ of *C. laurentii*. After treating grapefruits with a combined culture of the two agents, it was observed that, in comparison to the individual treatments using 1% bamboo vinegar or *C. laurentii* at a concentration of 10^7^ CFU mL^−1^, the BV-*C. laurentii* treatment group demonstrated a more effective capacity to control the spread of *P. digitatum* on grapefruits. Conversely, the *C. laurentii* treatment group exhibited superior inhibitory effects on the spread of *P. italicum* and *P. expansum* on grapefruits. Notably, both the BV and BV-*C. laurentii* treatment groups showed significant inhibitory impacts on the sporophore length of *P. expansum*. However, when it came to the grapefruit, the lesion diameters resulting from *P. expansum* - induced diseases in the BV and BV-*C. laurentii* treatment groups were significantly larger than those in the CK. This inconsistency between in vitro and in vivo results for *P. expansum* might be attributed to the fundamental differences in nutritional composition and physiological environment between the culture medium (PDB) and grapefruit tissue. Previous studies have demonstrated that the pathogenicity and secondary metabolism of *P. expansum* are strongly influenced by host-derived factors, such as sugar composition and concentration, organic acids, and other fruit-specific components (Kumar et al., 2017). Notably, the differential behavior of pathogens in culture media versus living fruit hosts has been well documented, with studies showing that the complex pathogen-host interaction significantly influences disease expression and cannot be fully replicated in vitro (Sanzani et al., 2013). Furthermore, recent research on yeast-fungus interactions has revealed that antifungal compounds effective in culture media may not necessarily exhibit the same efficacy on fruit, and vice versa Dor et al. (2021). Therefore, while BV and BV-*C. laurentii* treatments effectively inhibited *P. expansum* germ tube elongation in PDB medium, the more complex nutritional and physiological environment of grapefruit tissue may have attenuated this direct antifungal effect. Additionally, the competitive colonization dynamics between *C. laurentii* and *P. expansum* on the fruit surface, as well as the potential metabolism of bamboo vinegar components by fruit tissue, could further contribute to the observed discrepancies. These findings underscore the importance of validating in vitro observations with in vivo assays when evaluating biocontrol agents for postharvest applications.

CHI and GLU are pivotal enzymes associated with plant disease resistance, exhibiting direct antifungal activity (Dos Santos & Franco, 2023). CHI can hydrolyze the chitin in fungal cell walls, while GLU directly cleaves the β-1,3-glucan backbone of fungal cell walls, releasing oligosaccharides from the pathogen cell walls to induce plant defense responses (Virgen-Ortiz et al., 2020). The extracellular CHI secreted by *Saccharomyces cerevisiae* can inhibit the growth of *Aspergillus flavus*, causing deformation of its hyphae and thereby further enhancing the biocontrol activity of *S. cerevisiae* (Abdel-Kareem et al., 2019). Maluleke et al. (2022) discovered that CHI and GLU secreted by *Wickerhamomyces anomalus*, *Zygoascus meyerae*, and *Hyphopichia burtonii* can degrade the polysaccharides in the cell walls of *Botrytis cinerea* hyphae, effectively suppressing gray mold infection. Treatment with *Pichia guilliermondii* R13 significantly enhanced the activities of CHI and GLU in pepper fruits, accompanied by the accumulation of phytoalexins, which significantly reduced the lesion size of pepper anthracnose (Nantawanit et al., 2010). This study indicates that *C. laurentii* is capable of producing CHI and GLU. Following induction with bamboo vinegar, the activities of CHI and GLU in *C. laurentii* were significantly higher than those in the non-induced *C. laurentii* group. Moreover, on the fruit surface, the activities of CHI and GLU in both *C. laurentii* and BV-*C. laurentii* treatment groups were significantly enhanced. ROS serve as a pivotal indicator for evaluating the level of oxidative stress. During the processes of fruit softening and ripening, a substantial accumulation of ROS occurs (Ou et al., 2016). Nevertheless, excessive accumulation of ROS can lead to oxidative damage, triggering membrane lipid peroxidation within the cellular environment (Heil & Bostock, 2002). MDA, a key biomarker of membrane lipid peroxidation, reflects the extent of cell membrane damage and the oxidative stress status of the organism (Peng et al., 2025). GSH and GR collectively form the core of the antioxidant defense system in living organisms, maintaining the dynamic balance of reduced GSH within cells through synergistic actions, thereby protecting cells from oxidative damage (Sarvajeet et al., 2013). The results of this study show that after induction by bamboo vinegar, the GR activity of *C. laurentii* significantly increased, while the GSH content significantly decreased (Table 1). This seemingly contradictory result actually reveals that the cells are in a more efficient “prepared” state for antioxidant stress. The mechanism lies in that GR is the key enzyme that catalyzes the reduction of oxidized glutathione (GSSG) to reduced GSH. The significant increase in GR activity indicates that the potential of yeast cells to clear GSSG and regenerate GSH has been greatly enhanced. The ability to recycle glutathione enables cells to rapidly regenerate GSH and neutralize ROS in the face of sudden, intense oxidative stress (such as H_2_O_2_ treatment, Fig. 6), thereby demonstrating a stronger overall antioxidant capacity. At the same time, the significant decrease in MDA content in the BV-*C. laurentii* group (Table 1) directly confirms that the oxidative damage to the cell membrane is less, verifying the effectiveness of its antioxidant defense system. Therefore, the increase in GR activity and the decrease in GSH content (accompanied by lower MDA) jointly reflect that the antioxidant cycle system of *C. laurentii* has been activated after bamboo vinegar induction, placing it in a physiological state that can more quickly respond to and cope with oxidative stress. Total phenols and flavonoids act as direct antimicrobial agents to inhibit pathogen growth while scavenging excess ROS in plants, preventing oxidative damage to fruit cells and enhancing plant defense capabilities (Adil et al., 2024). Yan et al. (2023) demonstrated that the aqueous extract of *Ligusticum chuanxiong* could enhance the activities of CAT, GR, and SOD in yeast while reducing MDA levels, thereby protecting *Saccharomyces cerevisiae* from aging. Godana discovered that treatment of grapes with 1% chitosan-induced *P. anomala* not only enhanced POD, PAL, and PPO activities in grapes but also promoted the accumulation of total phenols and flavonoids, thereby strengthening the postharvest defense response of grapes against blue mold (Godana et al., 2020). PAL, recognized as the pivotal rate-limiting enzyme in phenylpropanoid biosynthesis, facilitates the deamination of *L*-phenylalanine to produce trans-cinnamic acid—a crucial precursor for lignin biosynthesis. Increased lignin content, as a key structural barrier component, effectively enhances cell wall thickness, thereby preventing pathogen invasion and improving fruit structural resistance (Wang et al., 2025). PPO catalyzes the oxidation of phenolic substances to generate quinone compounds, forming a physical-chemical double barrier at the site of plant injury, effectively blocking microbial colonization and spread (Zou et al., 2025). Sun et al. (2021) found that melatonin could enhance the colonization ability of *M. guilliermondii* Y-1 on apples, and their combined use significantly increased POD and PPO activities in apples, reducing the lesion diameter of gray mold by 35%. The findings from our research team earlier studies indicate that carboxymethyl chitosan, carboxymethyl cellulose, and melatonin can all induce *C. laurentii* to enhance the activities of antioxidant metabolism and phenylpropanoid metabolism enzymes in grapefruits, thereby effectively delaying grapefruit senescence and preserving fruit quality (Deng et al., 2021; Wang et al., 2019; Zhou et al., 2025). The results of this study reveal that bamboo vinegar can significantly increase GR activity in *C. laurentii*, reduce MDA and GSH levels, and enhance the antioxidant capacity and inherent disease resistance of *C. laurentii*. Subsequent verification on fruit demonstrated that BV-*C. laurentii* treatment significantly elevated the activities of PAL, PPO, CHI, and GLU in postharvest grapefruits, along with promoting the accumulation of total phenols and flavonoids. The enhanced activities of these defense-related enzymes and the enrichment of secondary metabolites collectively improved grapefruit resistance to green mold, extended shelf life, and reduced economic losses.

During post – harvest storage, fruits undergo a series of metabolic processes, with respiratory consumption and energy metabolism serving as the core processes throughout the entire post - harvest life cycle (Lin et al., 2024). The weight loss rate of fruits is a crucial parameter for evaluating the quality retention of post - harvested fruits (Liu et al., 2024). In our study, compared with the CK, the combined use of 1% bamboo vinegar and 10^7^ CFU mL^−1^ of *C. laurentii* significantly reduced water loss in grapefruits during storage. TA, RS, ASA, and TSS contents are currently the most commonly used physicochemical indicators for assessing changes in fruit quality during storage (Yu et al., 2021). *Wickerhamomyces anomalus* not only enhances the activities of antioxidant enzymes in tomatoes, including CAT, GR, glutathione peroxidase (GPX), and GST, but also delays tomato softening and water loss, maintain relatively high levels of TSS, TA, and vitamin C content, thereby improving the storage quality of tomatoes (Zhu et al., 2023). Tian et al. (2018) demonstrated that *Metschnikowia pulcherrima* effectively regulated mango fruit quality parameters, including peel coloration, firmness, total soluble solids, total acidity, and vitamin C content, thereby preserving postharvest storage quality. Notably, after the combined treatment with 1% bamboo vinegar and 10^7^ CFU mL^−1^ of *C. laurentii* in this paper, the contents of TA, RS, ASA, and TSS in grapefruits were all significantly higher than those in the CK. This indicates that the combined treatment with 1% bamboo vinegar and 10^7^ CFU mL^−1^ of *C. laurentii* can effectively maintain the fruit quality of grapefruits without any adverse effects. In terms of fruit appearance quality, color - difference parameters such as L*, *h*^*0*^, a*, b*, and c* are commonly used for evaluation, representing fruit brightness, hue angle, red - green value, blue - yellow value, and chroma, respectively (Shameena et al., 2024). Lu et al. (2014) found that the combined application of chitosan oligosaccharide (COS) and *Rhodosporidium paludigenum* could significantly enhance the resistance of citrus fruit to postharvest green mold. Compared with the treatments using *R. paludigenum* or COS alone, the combined treatment not only more effectively delayed the occurrence of the disease, but also significantly upregulated the expression levels of fruit defense-related genes (such as *CHI* and *PAL*), and exerted no adverse effects on the edible quality of citrus fruit. Overall, the individual use of 10^7^ CFU mL^−1^ of *C. laurentii*, 1% bamboo vinegar, and their combined use all had positive impacts on the fruit color of grapefruits. Among them, the combined treatment of 10^7^ CFU mL^−1^ of *C. laurentii* and 1% bamboo vinegar showed the best effect on maintaining appearance quality. This suggests that this combined treatment has no negative impact on the appearance quality during storage. The combined treatment effectively maintained the appearance quality, extended the shelf life, and reduced economic losses.

## 5 Conclusion

In conclusion, the combined treatment of 1% bamboo vinegar and 1 × 10^7^ CFU mL^−1^*C. laurentii* exhibits a dual effect of direct antibacterial action and induced resistance. It significantly inhibits the spore germination rate and the growth of spore tubes in *P. digitatum*, *P. italicum* and *P. expansum*. Concurrently, bamboo vinegar enhances GR activity of *C. laurentii*, reduces MDA and GSH contents, thereby strengthening its antioxidant capacity. Additionally, through the secretion of extracellular enzymes such as CHI and GLU, it directly degrades fungal cell walls, effectively suppressing the occurrence of green mold in grapefruits. Moreover, the combined treatment activates the defense enzymes PAL and PPO in grapefruits, elevates the contents of total phenols and flavonoids, and thus enhances the fruit's inherent disease resistance. Importantly, this treatment exerts no adverse effects on storage quality parameters, including firmness, TSS, RS, ASA, TA and color. Therefore, the combined treatment of 1% bamboo vinegar and *C. laurentii* offers a safe and efficient technological approach for green postharvest preservation of grapefruits, demonstrating significant potential as an alternative to chemical fungicides.

## CRediT authorship contribution statement

**Ruiqi Peng:** Writing – review & editing, Writing – original draft, Data curation. **Han Ye:** Writing – review & editing, Writing – original draft, Data curation. **Chunhua Wu:** Visualization, Methodology. **Xiahong He:** Conceptualization. **Fang Wang:** Writing – review & editing, Visualization, Methodology. **Jia Deng:** Writing – review & editing, Software, Methodology, Conceptualization.

## Fundings

This study was supported by the 10.13039/501100001809National Natural Science Foundation of China (NO. 32160394, 32460409); High-level Talents Training Support Program of Yunnan Province (YNWR-QNBJ-2020-205), Young and Middle-aged Academic and Technical Leaders Reserve Talent Program of Yunnan Province (202105AC160045), Joint Special Project for Agriculture of Yunnan Province (202301BD070001–155), Yunnan Revitalization Talent Support Program Young Talent Project (XDYC-QNRC-2022-0226), and National Modern Agricultural Industry Technology System Funded Project (CARS-21).

## Declaration of competing interest

The authors declare that they have no known competing financial interests or personal relationships that could have appeared to influence the work reported in this paper.

## Data Availability

Data will be made available on request.

## References

[bb0005] Abdelhai M.H., Awad F.N., Yang Q., Mahunu G.K., Godana E.A., Zhang H. (2019). Enhancement the biocontrol efficacy of *Sporidiobolus pararoseus* Y16 against apple blue mold decay by glycine betaine and its mechanism. Biological Control.

[bb0010] Abdel-Kareem M.M., Rasmey A.M., Zohri A.A. (2019). The action mechanism and biocontrol potentiality of novel isolates of *Saccharomyces cerevisiae* against the aflatoxigenic *aspergillus flavus*. Letters in Applied Microbiology.

[bb0015] Adil M., Filimban F.Z., Ambrin, Quddoos A., Sher A.A., Naseer M. (2024). Phytochemical screening, HPLC analysis, antimicrobial and antioxidant effect of *Euphorbia parviflora* L. (Euphorbiaceae Juss.). Scientific Reports.

[bb0020] Bhatta U.K. (2022). Alternative management approaches of *Citrus* diseases caused by *Penicillium digitatum* (green Mold) and *Penicillium italicum* (blue Mold). Frontiers in Plant Science.

[bb0025] Błaszczyk U., Wyrzykowska S., Gąstoł M. (2022). Application of bioactive coatings with killer yeasts to control post-harvest apple decay caused by *Botrytis cinerea* and *Penicillium italicum*. Foods.

[bb0030] Chen Y.P., Chen C., Wu H., He Q., Wu J., Yao J.Y., Chen F. (2025). Bamboo vinegar powder: Unveiling its antioxidant and antifungal efficacy through bioactive compound analysis and mechanistic insights. Food Chemistry.

[bb0035] Ciriminna R., Petri G.L., Angellotti G., Luque R., Fabiano Tixier A.S., Meneguzzo F., Pagliaro M. (2025). *Citrus* flavonoids as antimicrobials. Chemistry and Biodiversity.

[bb0040] Deng J., Li W., Ma D., Liu Y., Yang H., Lin J., Song G., Naik N., Guo Z., Wang F. (2021). Synergistic effect of carboxymethylcellulose and *Cryptococcus laurentii* on suppressing green mould of postharvest grapefruit and its mechanism. International Journal of Biological Macromolecules.

[bb0045] Devi P., Singh G., Suneja Y., Rattanpal H.S., Gupta M., Lhamu R. (2024). Potential value of bioactive and enzymatic antioxidant compounds in grapefruit (*Citrus* × *Paradisi Macf*.) varieties. Food Analytical Methods.

[bb0050] Dor S., Prusky D., Afriat-Jurnou L. (2021). Bacterial quorum-quenching lactonase hydrolyzes fungal mycotoxin and reduces pathogenicity of *Penicillium expansum*—Suggesting a mechanism of bacterial antagonism. Journal of Fungi.

[bb0055] Dos Santos C., Franco O.L. (2023). Pathogenesis-related proteins (PRs) with enzyme activity activating plant defense responses. Plants.

[bb0060] Edo G.S., Godana E.A., Ngolong Ngea G.L., Wang K., Yang Q., Zhang H. (2025). Improving biocontrol potential of antagonistic yeasts against fungal pathogen in postharvest fruits and vegetables through application of organic enhancing agents. Foods.

[bb0065] Feng M., Lv Y., Li T., Li X., Liu J., Chen X., Zhang Y., Chen X., Wang A. (2021). Postharvest treatments with three yeast strains and their combinations to control botrytis cinerea of snap beans. Foods.

[bb0070] Ganhão R., Estévez M., Morcuende D. (2011). Suitability of the TBA method for assessing lipid oxidation in a meat system with added phenolic-rich materials. Food Chemistry.

[bb0075] Garcia-Lor A., Bermejo A., Morales J., Hernández M., Medina A., Cuenca J., Navarro L., Aleza P. (2021). Strategies to produce grapefruit-like citrus varieties with a low furanocoumarin content and distinctive flavonoid profiles. Frontiers in Plant Science.

[bb0080] Godana E.A., Yang Q., Wang K., Zhang H., Zhang X., Zhao L., Guillaume Legrand N.N. (2020). Bio-control activity of *Pichia anomala* supplemented with chitosan against *Penicillium expansum* in postharvest grapes and its possible inhibition mechanism. LWT.

[bb0085] Gu N., Zhang X., Gu X., Zhao L., Godana E.A., Xu M., Zhang H. (2021). Transcriptomic and proteomic analysis of the mechanisms involved in enhanced disease resistance of strawberries induced by *Rhodotorula mucilaginosa* cultured with chitosan. Postharvest Biology and Technology.

[bb0090] Guan S., Yang F., Yao J., Liu C., Wang R., Ruan M., Yao Z., Liu C., Wan H., Li Z., Diao M., Cheng Y. (2025). Dynamic changes in volatile organic compounds of cherry tomato fruits during storage at different temperatures using HS-GC-IMS. Food Research International.

[bb0095] He Y., Degraeve P., Oulahal N. (2024). Bioprotective yeasts: Potential to limit postharvest spoilage and to extend shelf life or improve microbial safety of processed foods. Heliyon.

[bb0100] Heil M., Bostock R.M. (2002). Induced systemic resistance (ISR) against pathogens in the context of induced plant defences. Annals of Botany.

[bb0105] Huang J., Qin Y., Xie Z., Wang P., Zhao Z., Huang X., Chen Q., Huang Z., Chen Y., Gao A. (2023). Combined transcriptome and metabolome analysis reveal that the white and yellow mango pulp colors are associated with carotenoid and flavonoid accumulation, and phytohormone signaling. Genomics.

[bb0110] Huang Y., Cai Y., Yu T. (2022). Sodium glutamate as a booster: Inducing *Rhodosporidium paludigenum* to enhance the inhibition of *Penicillium expansum* on pears. Journal of Applied Microbiology.

[bb0115] Kozakiewicz G., Małajowicz J., Szulc K., Karwacka M., Ciurzyńska A., Żelazko A., Janowicz M., Galus S. (2025). The effect of a pectin coating with gamma-decalactone on selected quality attributes of strawberries during refrigerated storage. Coatings.

[bb0120] Kumar D., Barad S., Chen Y., Luo X., Tannous J., Dubey A., Glam Matana N., Tian S., Li B., Keller N., Prusky D. (2017). LaeA regulation of secondary metabolism modulates virulence in *Penicillium expansum* and is mediated by sucrose. Molecular Plant Pathology.

[bb0125] Li J., Li H., Ji S., Chen T., Tian S., Qin G. (2019). Enhancement of biocontrol efficacy of *Cryptococcus laurentii* by cinnamic acid against *Penicillium italicum* in citrus fruit. Postharvest Biology and Technology.

[bb0130] Li X., Yu L., An F., Bai H., Wisniewski M., Wang Z. (2023). Caffeic acid increases the fitness of *Candida oleophila* to the microenvironment of kiwifruit and its biocontrol performance against postharvest decay fungi. Postharvest Biology and Technology.

[bb0135] Li Z., Hao X., He T., Chen Y., Yang M., Rong C., Zheng X. (2024). Bamboo vinegar regulates the phytoremediation efficiency of *Perilla frutescens* (L.) Britt. By reducing membrane lipid damage and increasing cadmium retention. Journal of Hazardous Materials.

[bb0140] Lin Y., Chen Y., Zheng Y., Zhang H., Lin M., Wang H., Fan Z., Chen Y., Lin H. (2024). Energy and respiratory metabolism participate in dicyclohexylcarbodiimide and disodium succinate-mediated the alteration of energy status modulating pericarp browning of fresh longan. Postharvest Biology and Technology.

[bb0145] Lisboa H.M., Pasquali M.B., dos Anjos A.I., Sarinho A.M., de Melo E.D., Andrade R., Barros A. (2024). Innovative and sustainable food preservation techniques: Enhancing food quality, safety, and environmental sustainability. Sustainability.

[bb0150] Liu H., Song L., You Y., Li Y., Duan X., Jiang Y., Lu W. (2011). Cold storage duration affects litchi fruit quality, membrane permeability, enzyme activities and energy charge during shelf time at ambient temperature. Postharvest Biology and Technology.

[bb0155] Liu Z., Wang P., Liu Z., Sun L., Li X., Liu J., Xing R., Zhang X., Deng H., Zhang Y. (2024). Dynamic changes and correlation analysis of pumpkin quality and physiological indicators during postharvest storage. HortScience.

[bb0160] Lu L., Liu Y., Yang J., Azat R., Yu T., Zheng X. (2014). Quaternary chitosan oligomers enhance resistance and biocontrol efficacy of *Rhodosporidium paludigenum* to green mold in Satsuma orange. Carbohydrate Polymers.

[bb0165] Maluleke E., Jolly N.P., Patterton H.G., Setati M.E. (2022). Antifungal activity of non-conventional yeasts against *Botrytis cinerea* and non-Botrytis grape bunch rot fungi. Frontiers in Microbiology.

[bb0170] Nantawanit N., Chanchaichaovivat A., Panijpan B., Ruenwongsa P. (2010). Induction of defense response against *Colletotrichum capsici* in chili fruit by the yeast *Pichia guilliermondii* strain R13. Biological Control.

[bb0175] Nishimoto S., Koike S., Inoue N., Suzuki T., Ogasawara Y. (2017). Activation of Nrf2 attenuates carbonyl stress induced by methylglyoxal in human neuroblastoma cells: Increase in GSH levels is a critical event for the detoxification mechanism. Biochemical and Biophysical Research Communications.

[bb0180] Ou C., Liu Y., Wang W., Dong D. (2016). Integration of UV-C with antagonistic yeast treatment for controlling post-harvest disease and maintaining fruit quality of *Ananas comosus*. BioControl.

[bb0185] Peng R., Zhou A., Chen J., Wen M., Wang F., Wu J. (2025). Antifungal activity of *Bacillus velezensis* HY13 against anthracnose disease of *Buxus bodinieri* caused by *Colletotrichum fructicola*. Biological Control.

[bb0190] Pereyra M.M., Díaz M.A., Vero S., Dib J.R. (2024). Enhancing biological control of postharvest green mold in lemons: Synergistic efficacy of native yeasts with diverse mechanisms of action. PLoS One.

[bb0195] Reymick O.O., Liu D., Cheng Y., Ouyang Q., Tao N. (2022). Cuminaldehyde-induced oxidative stress inhibits growth of *Penicillium digitatum* in citrus. Postharvest Biology and Technology.

[bb0200] Sanzani S.M., Montemurro C., Di Rienzo V., Solfrizzo M., Ippolito A. (2013). Genetic structure and natural variation associated with host of origin in *Penicillium expansum* strains causing blue mould. International Journal of Food Microbiology.

[bb0205] Sarvajeet S.G., Naser A.A., Mirza H., Ritu G., Dipesh K.T., Iqbal A., Narendra T. (2013). Glutathione and glutathione reductase: A boon in disguise for plant abiotic stress defense operations. Plant Physiology and Biochemistry.

[bb0210] Shameena S., Lekshmi P.R.G., Gopinath P.P., Gidagiri P., Kanagarajan S. (2024). Dynamic transformations in fruit color, bioactive compounds, and textural characteristics of purple-fleshed dragon fruit (*Hylocereus costaricensis*) across fruit developmental stages under humid tropical climate. Horticulturae.

[bb0215] Shi Z., Deng J., Wang F., Liu Y., Jiao J., Wang L., Zhang J. (2019). Individual and combined effects of bamboo vinegar and peach gum on postharvest grey mould caused by *Botrytis cinerea* in blueberry. Postharvest Biology and Technology.

[bb0220] Strano M.C., Altieri G., Allegra M., Di Renzo G.C., Paterna G., Matera A., Genovese F. (2022). Postharvest technologies of fresh citrus fruit: Advances and recent developments for the loss reduction during handling and storage. Horticulturae.

[bb0225] Sun C., Huang Y., Lian S., Saleem M., Li B., Wang C. (2021). Improving the biocontrol efficacy of *Meyerozyma guilliermondii* Y-1 with melatonin against postharvest gray mold in apple fruit. Postharvest Biology and Technology.

[bb0230] Tian Y.q., Li W., Jiang Z.t., Jing M.m., Shao Y.z. (2018). The preservation effect of *Metschnikowia pulcherrima* yeast on anthracnose of postharvest mango fruits and the possible mechanism. Food Science and Biotechnology.

[bb0235] Virgen-Ortiz J.J., Morales-Ventura J.M., Colín-Chávez C., Esquivel-Chávez F., Vargas-Arispuro I., Aispuro-Hernández E., Martínez-Téllez M.A. (2020). Postharvest application of pectic-oligosaccharides on quality attributes, activities of defense-related enzymes, and anthocyanin accumulation in strawberry. Journal of the Science of Food and Agriculture.

[bb0240] Wang F., Deng J., Jiao J., Lu Y., Yang L., Shi Z. (2019). The combined effects of carboxymethyl chitosan and *Cryptococcus laurentii* treatment on postharvest blue mold caused by *Penicillium italicum* in grapefruit fruit. Scientia Horticulturae.

[bb0245] Wang F., Xiao J., Zhang Y., Li R., Liu L., Deng J. (2021). Biocontrol ability and action mechanism of *Bacillus halotolerans* against *Botrytis cinerea* causing grey mould in postharvest strawberry fruit. Postharvest Biology and Technology.

[bb0250] Wang H., Liu S., Wang H., Luo D., Yang C., Qi S., Wang M., Jia Y., Duan Y., Liu C., Chen Q. (2025). *GmLac55* enhanced soybean resistance against soybean cyst nematodes through lignin biosynthesis. International Journal of Molecular Sciences.

[bb0255] Wang J., Zhang B., Xun H., Yao X., Tang F. (2024). Simultaneous quantification of twelve compounds from bamboo/wood vinegar by gas chromatography-mass spectrometry. Separations.

[bb0260] Watanabe M., Igarashi K., Kato S., Kamagata Y., Kitagawa W. (2022). Critical effect of H_2_O_2_ in the agar plate on the growth of laboratory and environmental strains. Microbiology Spectrum.

[bb0265] Wu F., Wang H., Lin Y., Feng S., Li X. (2025). Biocontrol mechanisms of antagonistic yeasts on postharvest fruits and vegetables and the approaches to enhance the biocontrol potential of antagonistic yeasts. International Journal of Food Microbiology.

[bb0270] Wu H.y., Wang F., Yang L., Chen L., Tang J.r., Liu Y., Deng J. (2024). Carboxymethyl chitosan promotes biofilm-formation of *Cryptococcus laurentii* to improve biocontrol efficacy against *Penicillium expansum* in grapefruit. Advanced Composites and Hybrid Materials.

[bb0275] Wu Y., Lin H., Lin Y., Shi J., Xue S., Hung Y.C., Wang H. (2017). Effects of biocontrol bacteria *Bacillus amyloliquefaciens* LY-1 culture broth on quality attributes and storability of harvested litchi fruit. Postharvest Biology and Technology.

[bb0280] Yan Y., Zou Q., Zhou Y., He H., Yu W., Yan H., Yi Y., Zhao Z. (2023). Water extract from *Ligusticum chuanxiong* delays the aging of *Saccharomyces cerevisiae* via improving antioxidant activity. Heliyon.

[bb0285] Yang L., Ma D., Wang F., Liu L., Chen L., He X., Tang J., Deng J. (2025). Carboxymethyl cellulose-induced *Cryptococcus laurentii* improves disease resistance and regulates phenylpropane and reactive oxygen metabolism in grapefruit. Food Chemistry.

[bb0290] Yu K., Xu J., Zhou L., Zou L., Liu W. (2021). Effect of chitosan coatings with cinnamon essential oil on postharvest quality of mangoes. Foods.

[bb0295] Yu L., Peng Z., Dong L., Wang S., Ding L., Huo Y., Wang H. (2018). Bamboo vinegar powder supplementation improves the antioxidant ability of the liver in finishing pigs. Livestock Science.

[bb0300] Zhang W., Zhuang S., Gao J. (2025). Xylitol promotes the antioxidant and biocontrol efficiency of the antagonistic yeast, *Meyerozyma guilliermondii*. Frontiers in Microbiology.

[bb0305] Zhang X., Li B., Zhang Z., Chen Y., Tian S. (2020). Antagonistic yeasts: A promising alternative to chemical fungicides for controlling postharvest decay of fruit. Journal of Fungi.

[bb0310] Zhou A., Tang Y., Xia S., Wu H., Chen L., Tang J., He X., Deng J., Wang F. (2025). Postharvest application of melatonin-induced *Cryptococcus laurentii* suppresses blue mould in grapefruit by regulating cell wall and membrane lipid metabolism. LWT.

[bb0315] Zhou A., Wang F., Yin J., Peng R., Deng J., Shen D., Wu J., Liu X., Ma H. (2022). Antifungal action and induction of resistance by *Bacillus* sp. strain YYC 155 against *Colletotrichum fructicola* for control of anthracnose disease in *Camellia oleifera*. Frontiers in Microbiology.

[bb0320] Zhu M., Yang Q., Godana E.A., Huo Y., Hu S., Zhang H. (2023). Efficacy of *Wickerhamomyces anomalus* in the biocontrol of black spot decay in tomatoes and investigation of the mechanisms involved. Biological Control.

[bb0325] Zou H., Xiao Q., Li G., Wei X., Tian X., Zhu L., Ma F., Li M. (2025). Revisiting the advancements in plant polyphenol oxidases research. Scientia Horticulturae.

